# Quantitative and modularized CRISPR/dCas9-dCpf1 dual function system in *Saccharomyces cerevisiae*

**DOI:** 10.3389/fbioe.2023.1218832

**Published:** 2023-10-18

**Authors:** Qing Feng, Xiaoyu Ning, Lei Qin, Jun Li, Chun Li

**Affiliations:** ^1^ Key Laboratory of Medical Molecule Science and Pharmaceutics Engineering, School of Chemistry and Chemical Engineering, Ministry of Industry and Information Technology, Beijing Institute of Technology, Beijing, China; ^2^ Key Lab for Industrial Biocatalysis, Department of Chemical Engineering, Ministry of Education, Tsinghua University, Beijing, China

**Keywords:** *Saccharomyces cerevisiae*, CRISPR/dCas9-dCpf1, bifunctional system, quantitative, modular, β-carotene

## Abstract

**Introduction:** Both CRISPR/dCas9 and CRISPR/dCpf1 genome editing systems have shown exciting promises in modulating yeast cell metabolic pathways. However, each system has its deficiencies to overcome. In this study, to achieve a compensatory effect, we successfully constructed a dual functional CRISPR activation/inhibition (CRISPRa/i) system based on Sp-dCas9 and Fn-dCpf1 proteins, along with their corresponding complementary RNAs.

**Methods:** We validated the high orthogonality and precise quantity targeting of selected yeast promoters. Various activating effector proteins (VP64, p65, Rta, and VP64-p65-Rta) and inhibiting effector proteins (KRAB, MeCP2, and KRAB-MeCP2), along with RNA scaffolds of MS2, PP7 and crRNA arrays were implemented in different combinations to investigate quantitative promoter strength. In the CRISPR/dCas9 system, the regulation rate ranged from 81.9% suppression to 627% activation in the mCherry gene reporter system. Studies on crRNA point mutations and crRNA arrays were conducted in the CRISPR/dCpf1 system, with the highest transcriptional inhibitory rate reaching up to 530% higher than the control. Furthermore, the orthogonal CRISPR/dCas9-dCpf1 inhibition system displayed distinct dual functions, simultaneously regulating the mCherry gene by dCas9/gRNA (54.6% efficiency) and eGFP gene by dCpf1/crRNA (62.4% efficiency) without signal crosstalk.

**Results and discussion:** Finally, we established an engineered yeast cell factory for β-carotene production using the CRISPR/dCas9-dCpf1 bifunctional system to achieve targeted modulation of both heterologous and endogenous metabolic pathways in *Saccharomyces cerevisiae*. The system includes an activation module of CRISPRa/dCas9 corresponding to a gRNA-protein complex library of 136 plasmids, and an inhibition module of CRISPRi/dCpf1 corresponding to a small crRNA array library. Results show that this CRISPR/dCas9-dCpf1 bifunctional orthogonal system is more quantitatively effective and expandable for simultaneous CRISPRa/i network control compared to single-guide edition, demonstrating higher potential of future application in yeast biotechnology.

## 1 Introduction

*Saccharomyces cerevisiae* has been extensively studied and utilized as a model microbial platform for the efficient and sustainable production of biofuels, chemicals and natural products (Du et al., 2011; Nielsen and Keasling, 2016). While possessing the capability to generate a wide range of products through both endogenous and heterologous pathways, many engineered yeast cell factories struggle with low efficiency and individually regulated targets within intricate metabolic regulatory networks, significantly impeding the high-yield production of desired products. CRISPRa/i systems have become as a powerful approach of genome-wide engineering. The potency and effectiveness of programmable dCas-mediated gene activation or inhibition have been investigated by fusion of transactivation domains, modular RNA-protein interaction domains, and acting in synergy with co-factors for constructing complex transcriptional programs (Lian et al., 2014; Lian and Zhao, 2015). Multi-functional CRISPRa/i approaches have achieved significant progress in recent studies. A gRNA-tRNA array targeting 8 genes in yeast lipid networks resulted in a 30-fold increase in free fatty acid production (Zhang and Wang, 2019). An orthogonal CRISPRa/i system to simultaneous up- and downregulate the expression of mammalian genes have been builded and tested in a comprehensive comparison study (Martella et al., 2019). Three CRISPR systems of spCas9-saCas9 and enhanced Cas12a from Acidaminococcus have been evaluated to profile genetic interactions of multiple genes (Li et al., 2022) Developing efficient multiplexed combinatorial genomic engineering strategies remain a challenge for yeast cell factory optimization due to the sophistication of natural systems (Mccarty et al., 2020).

The eukaryotic promoter region is replete with structural domains, including cis-regulatory elements, introns, 5′-UTR, and 3′-UTR. Upon investigation, it has become evident that the structural domains within the core promoter region serve specific biological functions, underscoring the crucial importance of promoter region research in synthetic biotechnology (Dermitzakis et al., 2002; Thomas et al., 2003; Siepel et al., 2005; Martella et al., 2019). Recent studies have demonstrated that a promoter containing strong TATA box (TATATAAA) exhibits 2.56-fold higher activity than the one with weaker TATA box (CATTTAAA), and its activity is 4.9-fold higher than the promoters lacking any TATA box (Tang et al., 2020). Additionally, artificial chimeric intron promoters have been developed in *S. cerevisiae*; when ribosome-associated introns are used to regulate TDH3p promoter, gene expression levels can reach up to 50 times higher (Myburgh et al., 2020).

There are mainly six (I-VI) different types of CRISPR/Cas systems developed so far (Makarova et al., 2020), with particular emphasis on the CRISPR/Cas9 system (type II) and the CRISPR/Cpf1 system (type V), which have been extensively studied and widely applied. Common features shared by CRISPR/Cas9 and CRISPR/Cpf1 include the utilization of a CRISPR nuclease (either Cas9 or Cpf1) and rely on an individual complementary RNA (gRNA or crRNA) for the precise targeting of specific genes in genome editing (Cong et al., 2012; Gasiunas et al., 2012; Jinek et al., 2012; Wiedenheft et al., 2012). This encompassing capability covers gene deletion, insertion and mutation (Nishimasu et al., 2014; Fonfara et al., 2016; Ungerer and Pakrasi, 2016; Kim et al., 2017). Furthermore, gene activation (CRISPRa) and repression (CRISPRi) can be achieved at the transcriptional level using nuclease-inactivated dCas9 or dCpf1 proteins. When coupled with appropriately structured complementary RNAs and effector proteins, CRISPRa/i systems facilitate precise and multi-targeted transcriptional regulation.

The CRISPR/dCas9 system provides a versatile platform for investigating targeted transcriptional regulation. Through fusion with activators or repressors, dCas9 fusion proteins can be delivered to the regulatory region or coding region of any gene, allowing for precise and targeted regulation without causing DNA damage (Gilbert et al., 2014; Zalatan J. G. et al., 2015; La Russa and Qi, 2015; Deaner and Alper, 2017). Additionally, the gRNA-protein complex enables the design of multiple gRNAs to accurately regulate targeted DNA regions. However, it is worth noting that the length of gRNA used in the CRISPR/dCas9 system is typically long (>100 nt), necessitating the inclusion of the endonuclease Csy4 for processing gRNA arrays in multiplexed technologies (Yang and Li, 2017).

Compared to the CRISPR/dCas9 system, the CRISPR/dCpf1 system exhibits distinct advantages. These include a shorter crRNA (43 nt) for guiding dCpf1, the ability to target T-rich PAMs (5′-TTN-3′), the cleavage of pre-crRNAs to generate mature functional crRNAs without the need for trans-activating RNAs (tracrRNAs), and a smaller molecular mass of the dCpf1 protein. Recent literatures report that the CRISPR/dCpf1 system is more conducive to the simultaneous editing of multiple genes compared to the CRISPR/dCas9 system, demonstrating more sophisticated genome manipulation (Zhang et al., 2018; Nihongaki et al., 2019; Choi and Woo, 2020). Different dCas12a proteins constructed with synthetic transcriptional activators and repressors harnessing gene editing have been tested in *S. cerevisiae* (Yu and Marchisio, 2021). The transcriptional repression efficiencies could reach up to 95% in targeting 4 sgRNAs to three distinct genes in parallel in *S. cerevisiae* (McCarty et al., 2020). However, the CRISPR/dCpf1 system has its limitations: the lack of stem-loop structure of crRNA makes it less resistant to RNase molecules, resulting in overall weaker viability than the CRISPR/dCas9 system, which employs gRNAs with various stem-loops for modification (Zhang et al., 2018).

In this study, we investigated how each monofunctional system of CRISPR/dCas9 and CRISPR/dCpf1, could quantify selected yeast promoter strengths. We also explored the compatibility of various RNA scaffolds and different effector proteins to fabricate gene activation and inhibition. Then, we verified the orthogonality of CRISPR/dCas9 and CRISPR/dCpf1 systems in three engineered strains in *S. cerevisiae*. Finally, we established a β-carotene producing CRISPR/dCas9-dCpf1 bifunctional orthogonal system to flexibly redirect metabolic fluxes in the yeast cell. This system can be triggered by a gRNA-protein complex library and/or a crRNA array library in a quantitative and modularized manner, enabling fine-tuned multiplexed regulation of simultaneous activation and repression to modulate endogenous and exogenous metabolic pathways with no crosstalk.

## 2 Materials and methods

### 2.1 Strains, plasmids, and culture conditions

The strains, plasmids, and primers used in this study are listed in Supplementary Table S1. *S. cerevisiae* BY4741 strain was preserved by the research group. The Top10 competent strains of E.coli were purchased from Bomed Genetic Technologies Ltd., and the plasmids pESC-Ura and pESC-His used for plasmid vector construction were from the storage of the research group. Yeast Extract Peptone Dextrose Medium (YPD) contained 20 g/L peptone, 10 g/L yeast powder and 20 g/L glucose. Luria-Bertani (LB) medium contained 10 g/L peptone, 5 g/L yeast powder and 10 g/L sodium chloride. SD-Ura medium (uracil trap medium) contained 6.7 g/L yeast nitrogen source (YNB) without amino acids, 1.3 g/L other essential amino acids, histidine (His) 100 mg/L, leucine (Leu) 100 mg/L, tryptophan (Trp) 100 mg/L, 20 g/L glucose. SD-His medium (histidine deficient medium) contained 6.7 g/L YNB without amino acids and 1.3 g/L other essential amino acids. Without amino acid YNB 6.7 g/L, uracil (Ura) 50 mg/L, leucine (Leu) 100 mg/L, tryptophan (Trp) 100 mg/L, 20 g/L glucose. SD-Ura and SD-His media and pH was adjusted to 6.2 by 2 mol/L NaOH. Solid medium was prepared by adding 20 g/L Agar powder.

### 2.2 The method of SpdCas9 or FndCpf1 nuclease inactivation

Using plasmid PUC19-Cas9 as a template, primers were designed to split the SpCas9 gene into two fragments SpCas9(F) and SpCas9(R) and introduce mutations at the first and last ends to mutate the amino acid aspartic acid (D) at position 10 to alanine (A) and histidine (H) at position 841 to alanine (A), and the SpCas9(F) and SpCas9(R) fragments were ligated to obtain the SpdCas9 gene by OE-PCR, thus to realize SpdCas9 nuclease inactivation.

Using plasmid pY004-FnCpf1 as a template, primers were designed to split the FnCpf1 gene into two fragments FnCpf1(F) and FnCpf1(R) and mutations were introduced at the end to mutate the amino acid aspartate (D) at position 917 to alanine (A), and the FnCpf1(F) and FnCpf1(R) fragments were ligated by OE-PCR to obtain the FndCpf1 gene, thus to realize FndCpf1 nuclease inactivation.

### 2.3 gRNA plasmid library construction

The FBA1p/TEF1p promoter was amplified by PCR on the BY4741 genome. Taq DNA polymerase was purchased from TaKaRa (Dalian). Restriction endonuclease was purchased from Thermo Scientific. gRNA expression cassette was constructed on the basis of plasmid pRS423-gRNA. The target fragments SNR52p-20 bp and 20 bp-sgRN scaffold-SUP4t were obtained by PCR amplification. Plasmid pESC-Ura was cleaved with *Hind*III/*Kpn*I to obtain fragment pESC-Ura, and the three fragments were Gibson assembled by Gibson ligation. Again, the target fragments ADH1p-MCP-linker-VP64-ADH1t and ADH1p-MCP-linker-KRAB-ADH1t were obtained by PCR amplification. Plasmid pESC-Ura was digested with *Eco*RI/*Bam*HI to obtain fragment pESC-Ura, and the three fragments were subjected to Gibson assembly by Gibson ligation. Gibson assembly. The pESC-ura-PCP-VP64 (Ura-PV) and pESC-ura-PCP-KRAB (Ura-PK) expression vectors were obtained. The gRNA libraries were further transferred into *E. coli* Top10 and cultured on LB plates containing 100 μg/ml ampicillin. Extract the successfully transformed *E. coli* plasmid. The gRNA library was transferred to strain yDYW000 and cultured at 30°C in SC-Ura medium.

### 2.4 Yeast transformation and assembly

The fragments to be integrated on the *S. cerevisiae* genome in this study were transferred into *S. cerevisiae* cells by electrotransformation as follows. The yeast was transferred from the overnight culture to YPD liquid medium at a volume ratio of 1:10 (depending on the amount of transformation required) and incubated at 30°C for 4–5 h in a 200 rpm shaker. 1 ml of the bacterial solution was divided into 1.5 ml EP tubes and centrifuged at 5,000 rpm for 2 min. Resuspend the cells by adding 1 ml of distilled water to the EP tube, centrifuge at 5,000 rpm for 2 min, and discard the supernatant. Repeat. Add 40 μL of 1 M sorbitol solution to the EP tube, resuspend the cells by slow agitation with a gun tip, centrifuge at 5,000 rpm for 2 min, and discard the supernatant. Repeat. Resuspend with 50 μL of 1 M sorbitol solution. Add the fragments to be transformed to the EP tube at a molar ratio of 1:1 so that the amount of each fragment is not less than 1,000 ng. Mix well and add to a 2 mm electrotransformer cup, and after a 2,500 V shock, quickly add 1 ml of 1 M sorbitol medium to the electrotransformer cup, mix well by pipetting with a gun and aspirate into a clean EP tube. Incubate at 30°C for 1 h in a 200 rpm shaker. Centrifuge at 5,000 rpm for 2 min and discard the supernatant. Resuspend with 50 μL of 1 M sorbitol solution and spread evenly onto YPD solid medium with antibiotics and incubate at 30°C for 48 h.

### 2.5 Fluorescence intensity measurement

Recombinant yeast strains were precultured in the corresponding selective medium for 2 days and then inoculated into the fresh synthetic media with an initial OD of 0.1. Mid-log phase yeast cells were diluted 5-fold in ddH2O and eGFP an mcherry fluorescence signals were measured at 485–528 nm and 587–610 nm, respectively, using a Tecan Infinite M1000 PRO multimode reader (Tecan Trading AG, Switzerland). The fluorescence intensity (relative fluorescence units; RFU) was normalized to cell density that was determined by measuring the absorbance at 600 nm using the same microplate reader.

### 2.6 β-carotene production and quantification

β-Carotene producing strains with gRNAs were pre-cultured in SD-HIS-URA/G418 medium for approximately 2 days, inoculated into 20 mL of fresh medium with an initial OD600 of 0.1, and incubated for 5 days under aerobic conditions (30°C, 250 rpm). Y The yeast cells were collected, centrifuged at 13,000 g for 1 min, and the cell precipitate was resuspended in 1 ml of 3n HCl, boiled for 5 min and cooled in an ice bath for 5 min. The lysed cells were washed with ddH2O and resuspended in 400 μL of acetone to extract β-carotene. Cell debris was removed by centrifugation. The extraction step was repeated until the cell precipitate was white. Analyze the absorbance of the supernatant containing β-carotene at 454 nm.

### 2.7 gRNA and crRNA design

gRNAs and crRNAs for CRISPRa/i were designed using the CRISPOR tool (http://crispor.tefor.net/crispor.pybatchId=OZeMIBqmOOy55UwylOOJ) and those with high targeting and off-target scores were selected. Since there was no targeting score and off-target score for Cpf1, the following criteria were considered. gC content between 35% and 65%, no polyT, no secondary structure.

### 2.8 Quantitative real-time PCR analysis

Mid-log phase yeast cells were collected and used to determine the relative expression levels via qPCR. A total of 1 µg RNA samples were reverse transcribed into cDNA using a Transcriptor First Strand cDNA Synthesis Kit using an oligonucleotide dT primer (Roche, Indianapolis, Indiana, United States).

qRT PCR was performed using the 217 Applied Biosystems StepOnePlus Real-Time PCR System and the detaSYBR^®^ kit under the following reaction conditions: 95°C for 1 min, 45 cycles of 95°C for 15 s, 60°C for 15 s, 72°C 45 s, 95°C 10 s, 65°C 60 s, 97°C 1 s, 37°C 30 s. The standard internal reference gene is ACT1.

### 2.9 Data availability

The statistical analysis was carried out using one-way analysis of variance followed by Duncan’s multiple comparison tests. *p* values < 0.05 were considered statistically significant. The results are presented as the mean ± the standard deviation (SD) for a replication of *n* = 3.

## 3 Results

### 3.1 Quantitative promoter regulatory strength in CRISPR/dCas9 system

To investigate the quantified effects of promoter regulatory strength in the CRISPR/dCas9 system within *S. cerevisiae*, we selected two robust yeast promoters, FBA1 and TEF1, and employed mcherry as a reporter gene. Transcriptional regulator proteins VP64 (activator) and KRAB (inhibitor) were synthesized to create complexes with Sp-dCas9, serving as fusion proteins in dCas9-CRISPRa/i systems.

**FIGURE 1:**
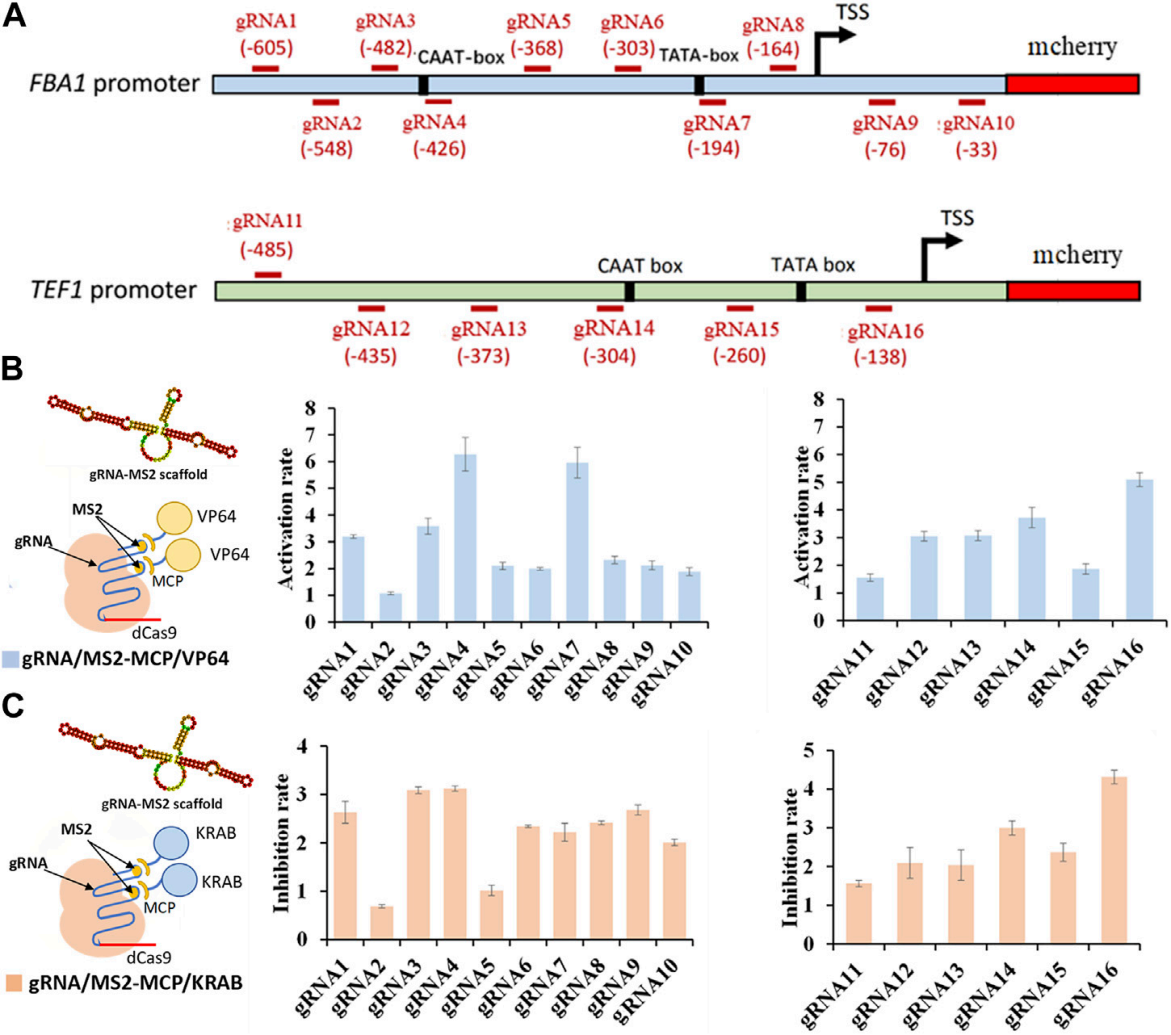
Regulation rate of targeted promoter sites by dCas9-CRISPRa/i systems. **(A)** Design of various gRNAs targeting FBA1 and TEF1 promoters. **(B)** Activation effect of CRISPRa (dCas9 incorporating activation effector of VP64 domain) on FBA1 promoter (RNA1-10) and TEF1 promoter (RNA11-16). **(C)** Inhibition effect of CRISPRi (dCas9 fused with inhibitory effector of KRAB domain) on FBA1 promoter (RNA1-10) and TEF1 promoter (RNA11-16). Promoter strength are represented by the fluorescence intensity of mcherry. All values are expressed as the means ± SDs (*n* = 3).

In total, we designed 16 gRNAs targeting distinct regions of the FBA1 and TEF1 promoters, with gRNA1-10 specific to FBA1 and gRNA11-16 dedicated to TEF1 (Figure 1A). The integration of gRNA scaffold MS2 with the activating effector VP64 yielded a 1 to 6.27-fold enhancement of mcherry gene expression (Figure 1B). Conversely, combining the MS2 gRNA scaffold with the repressor effector KRAB resulted in a 1 to 4.5-fold reduction of mcherry gene expression. In both scenarios, the most pronounced regulatory effects were observed in proximity to the TATA-box and CAAT-box elements (Figure 1C).

Conversely, combining the MS2 gRNA scaffold with the repressor effector KRAB resulted in a 1 to 4.5-fold reduction of mcherry gene expression. In both scenarios, the most pronounced regulatory effects were observed in proximity to the TATA-box and CAAT-box elements TATA-box, CAAT-box, and transcription start site (TSS). Notably, gRNA7 (-194) targeting TATA-box (TATA) of the FBA1 promoter, and gRNA16 (-138) targeting TATA-box (TAAAGATT) and TSS of TEF1 promoter display significant stronger regulation in mcherry expression. In addition, the higher regulation rates caused by gRNA4 (-426) targeting CAAT-box (CAAT) of FBA1 promoter and gRNA14 (-304) targeting CAAT-box (TGAT) of TEF1promoter, were attributed to their closer proximity to the cis-acting elements within the promoter core region (Figures 1B, C).

### 3.2 Compatibility of gRNA scaffold and effector proteins in CRISPR/dCas9 system

**FIGURE 2:**
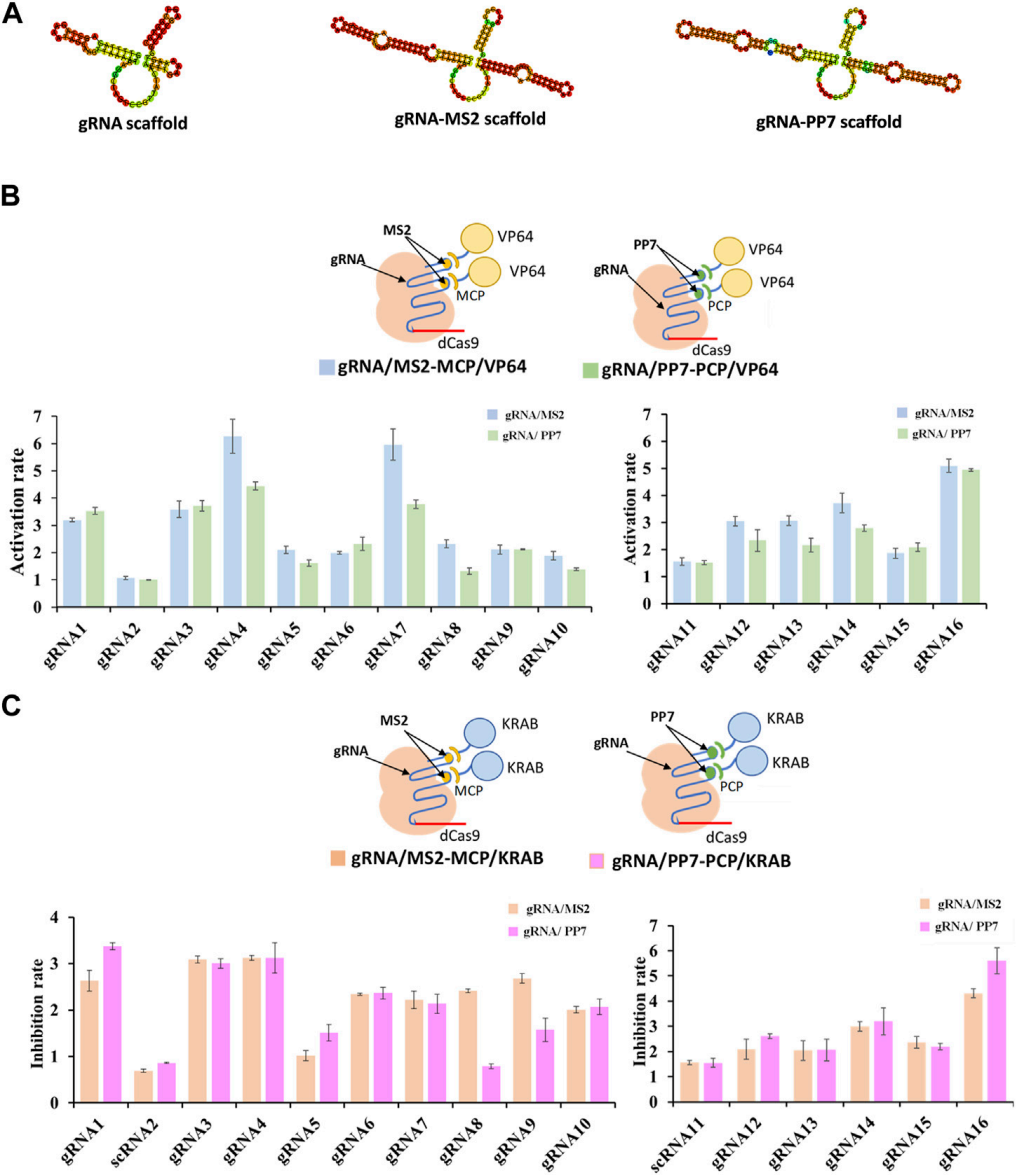
Compatibility of various gRNA scaffolds in dCas9-CRISPRa/i systems. **(A)** Representative secondary structure of gRNA scaffold, gRNA-MS2 scaffold and gRNA-PP7 scaffold. **(B)** Schematic representation of complexes of dCas9 protein guided by gRNA-MS2 recruiting activating effector proteins of VP64, and the corresponding activation rates tested by the fluorescence intensity of mcherry. **(C)** Schematic representation of complexes of dCas9 protein guided by gRNA-PP7 recruiting inhibiting effector proteins of KRAB, and the corresponding inhibition rates tested by the fluorescence intensity of mcherry. All values are expressed as the means ± SDs (*n* = 3).

The crucial component within the CRISPR/dCas9 system, responsible for identifying target genes and regulating dCas9 protein targeting, is known as the guide RNA, or gRNA. In efforts to achieve precise transcriptional regulation, certain researchers have integrated RNA-binding proteins (RBPs) fused with various effector proteins into the guide RNA sequence (Zalatan et al., 2015). The establishment of RNA-protein complexes followed a systematic procedure. Initially, RNA scaffolds, either MS2 RNA scaffold or PP7 RNA scaffold, were incorporated into the four-loop and stem-loop regions of selected guide RNAs (Figure 2A). Subsequently, MCP (MS2 capsid protein) or PCP (PP7 capsid protein) was fused with different effector proteins, namely, VP64 for activation (CRISPRa) and KRAB for inhibition (CRISPRi), forming the dCas9-gRNA complex. The results indicated that the gRNA/MS2-MCP/VP64 complex demonstrated higher activation rates in transcriptional regulation compared to the gRNA/PP7-PCP/VP64 complex (as shown in gRNA4/MS2, gRNA7/MS2, gRNA12/MS2, gRNA13/MS2, gRNA14/MS2) (Figure 2B). Conversely, stronger inhibition rates were observed in the gRNA/PP7-PCP/KRAB complex compared to the gRNA/MS2-MCP/KRAB complex when considering the gRNA scaffold and effector protein combinations (shown in gRNA1/PP7, gRNA3/PP7, gRNA4/PP7, gRNA16/PP7) (Figure 2C).

**FIGURE 3:**
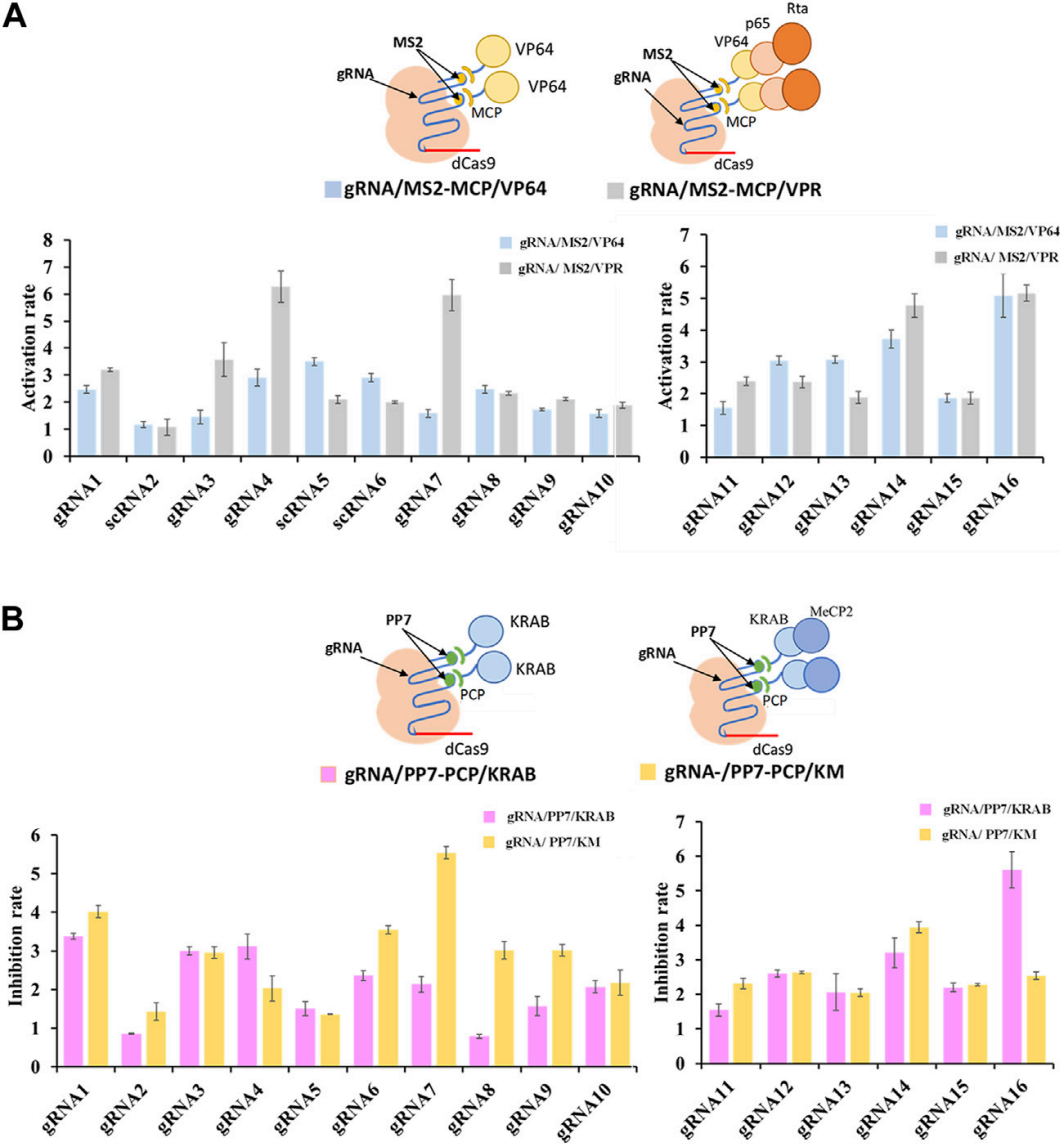
Compatibility of various effector proteins in dCas9-CRISPRa/i systems. **(A)** Schematic representation of complexes of dCas9 protein guided by gRNA-MS2 recruiting activating effector proteins of VP64 or VP64-P65-Rta, and the corresponding activation rates tested by the fluorescence intensity of mcherry. **(B)** Schematic representation of complexes of dCas9 protein guided by gRNA-PP7 recruiting inhibiting effector proteins of KRAB or KRAB-MeCP2, and the corresponding inhibition rates tested by the fluorescence intensity of mcherry. All values are expressed as the means ± SDs (*n* = 3).

To further enhance the regulatory potential of the CRISPR/dCas9 system, multiple effector proteins VP64-p65-Rta (VPR) and KRAB-MeCP2 (KM), were introduced. Both combinations exhibited more potent regulation rates compared to single effector protein. The multiple effector complex of gRNA/MS2-MCP/VPR significantly increased transcriptional activation up to 5.5-fold (shown in gRNA7) in contrast to the single effector complex of gRNA/MS2-MCP/VP64 (Figure 3A). Similarly, the complex of gRNA/PP7-PCP/KM increased transcriptional inhibition up to 2.6-fold (shown in gRNA7/PP7) compared tothe single effector complex of gRNA/PP7-PCP/KRAB (Figure 3B). Overall, multiplexed RNA-protein complexes in the CRISPR/dCas9 system yielded more efficient regulatory rates for both CRISPRa and CRISPRi. Interestingly, concerning the compatibility of gRNA scaffolds and effector proteins within the CRISPR/dCas9 system, we observed that the MS2-MCP pair exhibited better compatibility with activator proteins (VP64, p65, Rta), while the PP7-PCP pair demonstrated better compatibility with repressor protein (KRAB, MeCP2) in transcriptional regulations.

### 3.3 Quantitative promoter regulatory strength in CRISPR/dCpf1 system

**FIGURE 4:**
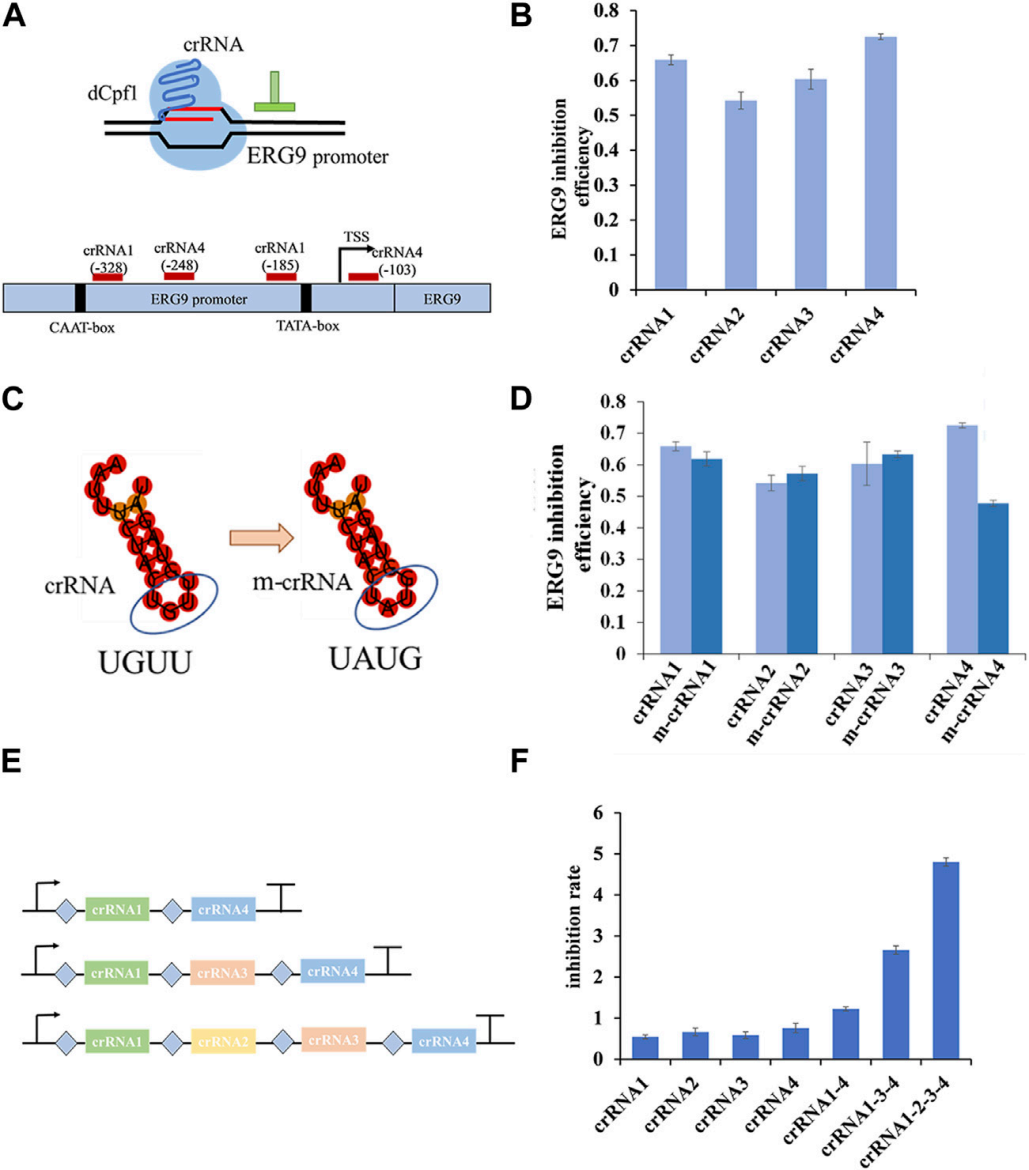
Regulation rate of targeted promoter and crRNAs in dCpf1-CRISPRi systems. **(A,B)** Design of crRNAs targeting ERG9 promoter regions and their corresponding inhibition efficiency in transcription level. **(C,D)** Schematic representation of crRNA scaffolds modification of crRNA (UGUU) into m-crRNA (UAUG), and their corresponding inhibition effect of ERG9 gene expression. **(E,F)** Design of crRNA arrays (connected by linkers) and the corresponding inhibition rates of targeting ERG9 promoter. All values are expressed as the means ± SDs (*n* = 3).

To investigate the regulatory effect of CRISPR/dCpf1 system, we designed four crRNAs targeting the yeast ERG9 promoter region based on an analysis of its transcription start site (TSS), TATA-box, and CAAT-box using CRISPR-direct software and RNA-fold software (Figure 4A). RT-qPCR results demonstrates efficient inhibition rates of ERG9 gene ranged from 54.2% (crRNA2) to 72.5% (crRNA4) (Figure 4B).

In the CRISPR/dCpf1 system, the short (∼20 bp) crRNA secondary structures often show only a single loop region, which is not conducive to constructing complicated RNA scaffold. As shown in Figure 4C, the DR scaffold loop region of the four selected crRNAs targeting the ERG9 promoter was mutated from UGUU to UAUG (ecrRNA). However, these mutations of the DR loops of crRNA1, crRNA2 and crRNA3 did not enhanced the inhibition rate in CRISPR/dCpf1 system. In fact, the inhibition rate of ecrRNA4 reduced from 72.5% to 47.8% when compared to that of crRNA4 (Figure 4D).

Therefore, several crRNA arrays were designed to further quantify the regulatory effect of ERG9 promoter (Figure 4E). The CRISPR/dCpf1 system can directly employ the Fn-dCpf1 protein to cleave crRNA arrays and subsequently utilize multiple crRNAs to simultaneously target multiple sites, enabling multiplex genome editing. All three multiplex crRNAs (crRNA1-4, crRNA1-3-4, and crRNA1-2-3-4) have displayed significantly higher transcriptional inhibitory rates (60%, 240%, and 530%) comparing with single crRNAs (Figure 4F).

### 3.4 Construction of CRISPR/dCas9-dCpf1 orthogonal system

**FIGURE 5:**
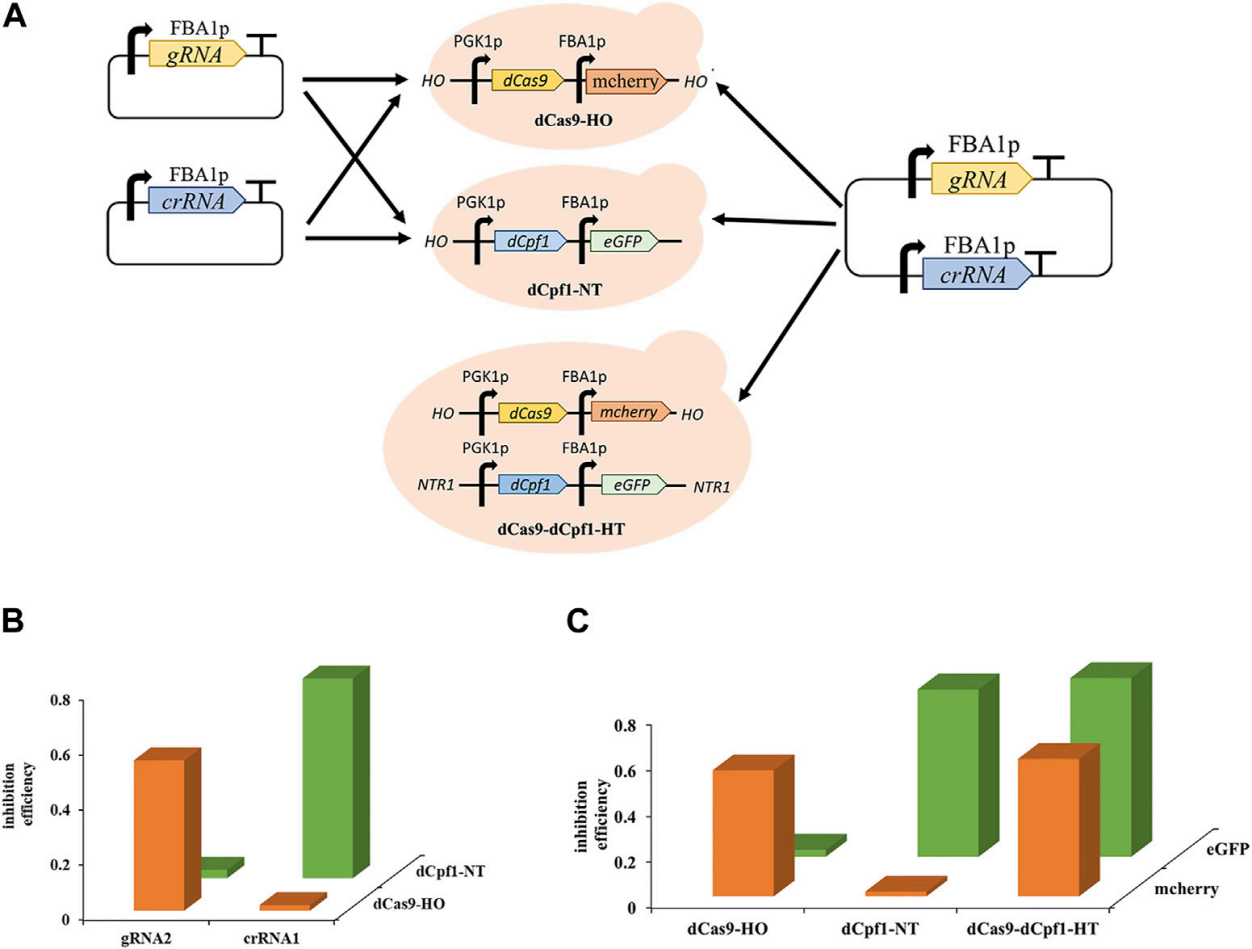
Orthogonality of CRISPR/dCas9-dCpf1. **(A)** Design of CRISPR single system and orthogonal CRISPRa/i system based on eGFP expression. **(B)** The orthogonality was tested by co-transforming the different CRISPR proteins (dCas9, dCpf1) and gRNA/crRNA with different origins. **(C)** CRISPR/dCas9-dCpf1 dual system, it has effective inhibition in both mcherry and eGFP, the dCas9 system inhibited only mcherry, and the dCpf1 system inhibited only eGFP. All values are expressed as the means ± SDs (*n* = 3).

The development of multifunctional CRISPRa/i systems has enabled versatile regulation using orthogonal dCas proteins. Orthogonal Cas proteins, sourced from different bacterial species, exclusively recognize their corresponding complementary RNAs and nd necessitate distinct PAM sequences for targeting. The dCas9 protein can only be guided by its complementary gRNAs and dCpf1 proteins can only respond to its complementary gRNAs, while dCpf1 proteins are responsive solely to their complementary crRNAs due to the recognition of distinct PAM sequences, with the dCas9 system recognizing cytosine-rich PAM sequences and the dCpf1 system recognizing thymine-rich PAM sequences. To assess the orthogonality of Sp-dCas9 and Fn-dCpf1 proteins, experiments were conducted in two separate yeast strains, namely, dCas9-HO and dCpf1-NT. These strains had the dCas9 gene integrated into the HO site of the genome and the dCpf1 gene integrated into the NTR1 site within the *S. cerevisiae* genome. Upon introducing complementary gRNAs and crRNAs targeting the FBA1 promoter into these yeast strains, the dCas9-bound gRNAs effectively induced transcriptional inhibition of the reporter gene eGFP, with a 54.6% repression rate for gRNA2 observed exclusively in the dCas9-HO strain, while the dCpf1-bound crRNAs efficiently mediated transcriptional inhibition of the reporter gene eGFP, yielding a 72.5% repression rate for crRNA2, exclusively in the dCpf1-NT strain (Figures 5A, B).

Furthermore, to establish a CRISPR/dCas9-dCpf1 orthogonal system, an engineered yeast strain named dCas9-dCpf1-HN was created. This strain incorporated genes encoding dCas9 and mcherry, and dCpf1 and eGFP, inserted into either the HO or NTR1 site of the genome, respectively (Figure 5A). Plasmids containing both gRNA and crRNA sequences targeting the FBA1 promoter were introduced into the aforementioned three engineered yeast strains: dCas9-dCpf1-HN, dCas9-HO, and dCpf1-NT. The fluorescence results for the reporter genes, mcherry and eGFP (both driven by FBA1 promoters), exhibited clear orthogonality in regulatory patterns. Specifically, the dCas9-HO strain demonstrated 55.6% effective inhibition exclusively in mcherry (corresponding to gRNAm) and not in eGFP (corresponding to crRNAe), while the dCpf1-HO strain displayed 73.5% effective inhibition exclusively in eGFP (corresponding to crRNAe) and not in mcherry (corresponding to gRNAm). In the case of the dCas9-dCpf1-HN strain, it exhibited effective inhibition in both mcherry and eGFP, without crosstalk between the two dCas systems. Notably, the inhibition rates of the dCas9-dCpf1 co-expressing system (60.3% for eGFP, 78.6% for mcherry) closely mirrored those of the single Cas protein expressing systems (55.6%, 73.5%) (see Figure 5C). Thus, the CRISPR/dCas9-dCpf1 dual system has been successfully validated for gene regulation in *S. cerevisiae*.

### 3.5 CRISPR/dCas9-dCpf1 bifunctional system in β-carotene production

**FIGURE 6:**
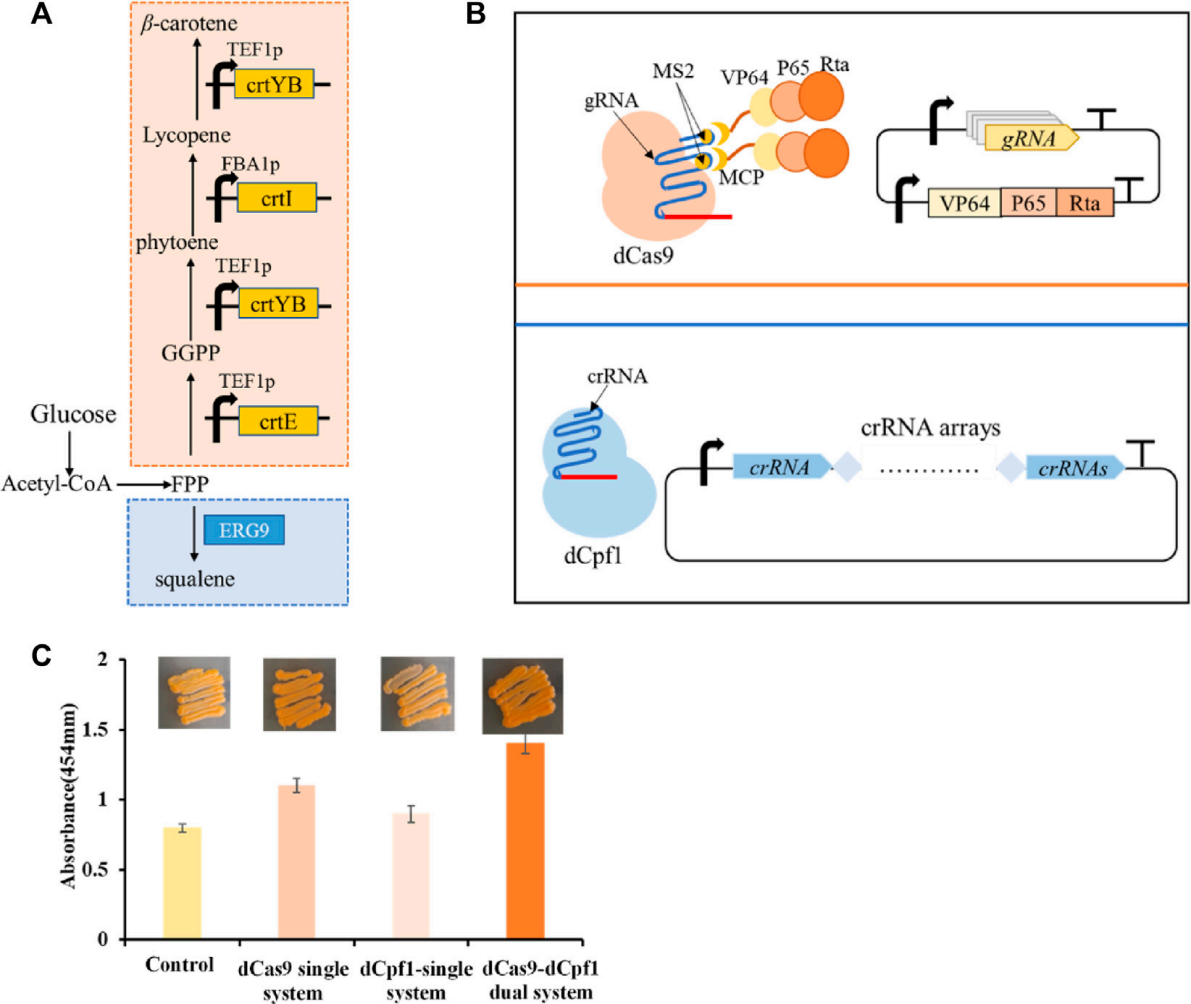
β-carotene metabolism in a bifunctional CRISPR/dCas9-dCpf1 system. **(A)** β-carotene metabolic pathway and ERG9 Competitive pathway. **(B)** The constructed CRISPRa/dCas9 module and CRISPRi/dCpf1 module respectively act on the β-carotene metabolic pathway and ERG9 competitive pathway. **(C)** The fold change calculated by light absorbance at 454 nm quantifies shows the yields of β-carotene in the following four systems, in the unregulated system, the dCas9 single system where only CRISPRa module is implemented, in the dCpf1 single system where only CRISPRi module is implemented, and in the CRISPR/dCas9-dCpf1 dual system where both CRISPRa and CRISPRi modules are activated. All values are expressed as mean ± standard deviation (*n* = 3).

To study the application of CRISPR multifunctional system in terpenoid yeast cell factories, the β-carotene pathway is commonly employed for validation (Naseri et al., 2019). The construction of the β-carotene metabolic pathway in the engineered dCas9-dCpf1-HN yeast strain necessitates the integration of three heterologous genes—CrtE, CrtI, and CrtYB—under the control of FBA1 and TEF1 promoters into the YPRC3 single-copy locus within the genome. This integration forms the basis for constructing a CRISPR/dCas9-dCpf1 bifunctional system. The newly developed β-carotene-producing yeast strain, named dCas9-dCpf1-Carotene, comprises two integral modules: the CRISPRa/dCas9 module and the CRISPRi/dCpf1 module (Figures 6A, B). The CRISPRa/dCas9 module operates in conjunction with a library containing 136 plasmids (CP1 to CP136), each harboring distinct combinations of gRNA-protein complexes designed to finely regulate the expression of crtE, crtI, and crtYB genes. The multiplexed combinations within the plasmid library are detailed in Table 1, featuring the previously mentioned 16 gRNAs, each possessing varying regulatory intensities and targeting the FBA1 and TEF1 promoters. These gRNAs are customized with either MS2 or PP7 RNA scaffolds, and the RNA-binding proteins MCP and PCP are fused with activating effector proteins such as VP64, VP64-p65-Rta (VPR), or repressing effector proteins such as KRAB, KRAB-MeCP2 (KM). In the CRISPRi/dCpf1 module, a small library of crRNA arrays was constructed to downregulate the ERG9 gene expression in the competing pathway. Each module can be activated through the application of the above stated plasmid libraries.

**TABLE 1:** dCas9-gRNAs (16 gRNAs targeting FBA1 and TEF1 promoter region) 136 combination of gRNAs with scaffolds and effector proteins.

	gRNA		Protein		β-carotene
Plasmids	gRNA	gRNA Scaffold	RNA Binding Protein	Effector Protein	Colored results
CP1-16	gRNA 1-16	MS2	MCP	VP64	
CP17-32				VP64-P65-Rta	
CP33-48				KRAB	
CP49-64				KRAB-MeCP2	
CP65-80	gRNA 1-16	PP7	PCP	VP64	
CP81-96				VP64-P65-Rta	
CP97-112				KRAB	
CP113-128				KRAB-MeCP2	
CP129-130	gRNA6-gRNA14	MS2	MCP	VP64	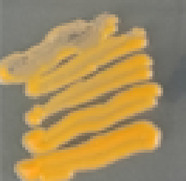
CP131-132				VP64-P65-Rta	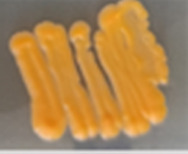
CP133-134	gRNA3-gRNA12	PP7	PCP	VP64	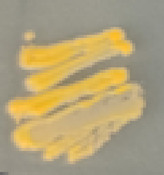
CP135-136				VP64-P65-Rta	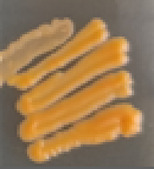

The highest β-carotene yield, quantified via light absorbance at 454 nm, was observed in the complex of gRNA6-gRNA14/MS2-MCP/VP64-P65-Rta, targeting the heterologous pathway within the CRISPRa/dCas9 module, and the complex of crRNA1-2-3-4 targeting the endogenous pathway within the CRISPRi/dCpf1 module. This resulted in a production yield 85% higher than that of the control. In comparison to individual monofunctional systems, the CRISPR/dCas9-dCpf1 dual system exhibited a 45% increase in efficiency over the dCas9 system alone and a 65% increase in efficiency over the dCpf1 system alone. Thus, we have successfully established a CRISPR/dCas9-dCpf1 bifunctional system for precise regulation of the heterologous β-carotene pathway and the competing endogenous ERG9 pathway (Figure 6C). In contrast to monofunctional CRISPR/dCas9 systems, the CRISPR/dCas9-dCpf1 dual system not only reduces crosstalk resulting from simultaneous activation and inhibition by gRNAs but also enables modular and quantitative metabolic engineering within *S. cerevisiae*.

## 4 Discussion

The CRISPR/dCas9 technology has witnessed rapid advancements, establishing itself as a versatile tool for genome manipulation. In parallel, the CRISPR/dCpf1 system has emerged as a potent alternative, demonstrating superior potential for transcriptional regulation when compared to the dCas9 system (Zhang et al., 2018; Choi and Woo, 2020; Yu and Marchisio, 2021). Each CRISPRa/i system boasts its unique set of advantages and limitations. Our primary objective in this study is to conduct a comprehensive exploration and harness the diverse attributes and potentials of both platforms. We aim to develop a quantified, multiplexed orthogonal gene activation and repression system employing CRISPR/dCas9 and CRISPR/dCpf1 within *S. cerevisiae*.

Extensive studies on yeast promoters have unveiled a notable trend in CRISPR/dCas9-mediated transcriptional activation. This trend reveals a progressive increase in activation intensity from the distal regions towards the core promoter, with maximal activation occurring withinthe core promoter region (Deaner and Alper, 2017). Research that analyzed 96 gRNAs shows that the best transcriptional regulation sites are in the 200 bp region upstream of the transcription start site (TSS) (Konermann et al., 2015). We have tested designed gRNAs targeting FBA1 and TEF1 promoters and have found out that the highest regulation sites were close to their TATA-box and CAAT-box both in activation and inhibition in CRISPR/dCas9 system. On the contrary, we did not discover similar regulatory pattern of crRNAs in the CRISPR/dCpf1 system targeting ERG9 promoter, even though the transcriptional repression efficiency of single crRNA could reach as high as 72.5%. Consequently, distinct complementary RNA design strategies, accounting for promoter location constraints, are warranted within these two systems.

The gRNA, serving as a pivotal structural element in the CRISPR system, has been subjected to extensive exploration. In melanoma models, the addition of the MS2 aptamer sequence to the gRNA tetra-loop or stem-loop facilitated the recruitment of the ligand-protein MCP fused with the VP64 activation domain, resulting in a noteworthy 12-fold increase in transcriptional activation was obtained compared to dCas9-VP64. Our study also delves into the pairing patterns of MS2 and PP7 aptamers with RNA binding protein (RBP) adaptors, elucidating their collaborative interaction with activating and repressing effector proteins (Mali et al., 2013; Zalatan et al., 2015). It is worth noting that the currently available RNA aptamers remain somewhat limited. In our work, we have similarly employed the MS2 and PP7 RNA scaffolds, along with their corresponding RBPs, MCP and PCP, to construct RNA-protein complexes for activation or repression within the CRISPR/dCas9 system. Our results show that the MS2-MCP pairing exhibits superior compatibility with activator proteins such as VP64 and VP64-p65-Rta, while the PP7-PCP pairing demonstrates enhanced synergy with repressor proteins like KRAB and KRAB-MeCP2 during transcriptional regulations. It's important to highlight that this pairing effect deviates somewhat from Martella’s findings (Martella et al., 2019). In addition, we have designed eight distinct sets of gRNA scaffold and RBP-effector pairs to build up a library with over one hundred possibilities for quantitative fine-tuning of pathway of interest, even though the β-carotene yield increase is not satisfying comparing with other’s work. For example, a gRNA-tRNA array in CRISPR/Cas9 was reported for multiplexed engineering resulting in a remarkable 30-fold increase in free fatty acid production in *S. cerevisiae* (Zhang and Wang, 2019).

In contrast, within the CRISPR/dCpf1 system, the dynamics of crRNA structure modifications reveal a different landscape. Previous finding shows that alterations to the loop region of the crRNA scaffold can lead to reduced or even complete loss of Cpf1 protein activity, although in rare instances, Cpf1 nuclease activity might be enhanced (Teng et al., 2019). Similarly, our efforts to mutate the DR scaffold loop region of crRNAs did not result in an improved repression efficiency of the CRISPR/dCpf1 system on the ERG9 promoter. This suggests that the structural changes induced by mutated crRNAs might have affected their affinity for directing dCpf1 protein. However, the utilization of crRNA arrays in our experiments demonstrated significantly increased efficiency.

Crosstalk among multi-activator proteins can enhance chromatin condensation and accumulation of histone marks by enabling the recruitment of more chromatin modifiers to target gene transcription (Chavez et al., 2015). CRISPRa technologies have attempted to use multiple activator domains fused to dCas9 directly, through a protein scaffold (e.g., VP64 fused to superfolder GFP) or through an RNA scaffold (e.g., p65 and HSF1fused to MCP) to perturb gene expression in mammalian cells (Martin, 2017). CRISPRi based on dCas9 can repress transcription directly, but it is more efficient when dCas9 is fused to repressor domains including KRAB, CS (chromoshadow), Mxi1, WPRW and SID4X to potentiate regulatory inhibition in mammalian cells (Dominguez et al., 2016). Our strategy to improve the transcriptional efficiency in CRISPR/dCas9 system is also to enable multiple effector proteins of VP64-p65-Rta (VPR) for stronger activation and KRAB-MeCP2 (KM) for stronger repression. In CRISPR/dCpf1 system, reports have shown that effector proteins of VP64, p65, or KRAB can also be fused to dCpf1 for transcriptionally genes regulations (Nihongaki et al., 2010; Yeo et al., 2018). In the future, the co-localization of multiple effector domains for the promotion of heterochromatin and histone methylation could potentially further enhance regulatory efficiency within n the CRISPR/dCpf1 genome editing system.

Multiplexed CRISPR technologies have benefited applications on large-scale genome engineering and the rewiring of metabolic pathways. Notably, gRNA (MS2)-SoxS for transcriptional activation and gRNA-mediated transcriptional repression was used to establish a bifunctional CRISPR-dCas9 system in bacteria (Dong et al., 2019). While literature exploring dCpf1-involved orthogonal genome engineering remains relatively limited, recent efforts have led to the development of Cas9 and Cas12a orthogonal gene manipulation systems employing fusion guide RNAs (gRNAs) in human cells (Shin et al., 2020). Emerging techniques based on Cas12a/Cas13a or Cas12a/Cas9 systems have emerged to orchestrate genetic reprogramming (Li et al., 2022; Zhu et al., 2022; Tian et al., 2022). However, as the trend towards multiplexed approaches gains momentum, questions arise concerning the effectiveness of gRNA arrays in achieving desired effects, given that each gRNA is context-dependent (Mccarty et al., 2020). As such, our efforts have been dedicated to the quantitative evaluation of gRNAs targeting two commonly used yeast promoters, as well as combinations of gRNAs and effectors within their native contexts. Our overarching strategy is to develop an orthogonal CRISPR/dCas9-dCpf1 bifunctional system, with the aim of achieving simultaneous quantitative upregulation (dCas9) and downregulation (dCpf1). This endeavor is poised to enrich the future application of CRISPR systems in metabolic networks.

Notably, Zhao’s group pioneered the construction of an orthogonal tri-functional CRISPR system, known as CRISPR-AID, in *S. cerevisiae*. Their innovative approach involved orthogonal and functional validation of multiple Cas proteins, resulting in a remarkable 2.8-fold increase in β-carotene production. This achievement was primarily attributed to the upregulation of HMG1, downregulation of ERG9, and the deletion of ROX1, with a predominant focus on endogenous upstream metabolic pathways (Lian et al., 2017). A similar study could be found in programmable control over multiple genes with simultaneous activation and repression in bacteria (Dong et al., 2019). However, when implementing heterologous pathways, the need for dynamic control becomes evident, as these pathways mayproduce toxic intermediates to affect cellular metabolism. Hence, the CRISPR/dCas9-dCpf1 bifunctional system developed in our study is designed to exert direct and quantitative regulation over the pathway of interest in a dynamic fashion. This system targets the heterologous β-carotene-producing genes (crtE, crtI, and crtYB) by leveraging CRISPR-dCas9, which is achieved by harnessing the gRNA library derived from the promoters of FBA1 and TEF1p, in conjunction with RNA scaffolds and effector proteins. The quantitative insights gained from the study of these promoters hold the potential for dynamic regulation and the rapid optimization of other heterologous metabolic pathways to achieve desired phenotypes. Furthermore, our work employs the strategy to separate the regulation of heterologous pathway from native pathways in a modular manner. We have assigned the dCas9 activation module with heterologous product producing pathway, and the dCpf1 inhibition module with the yeast endogenous competing pathway separately by using the orthogonality of the CRISPR/dCas9-dCpf1 bifunctional system to minimize the potential toxic cellular effect by heterologous intermediates. Hopefully it could broaden the utility of CRISPR/Cas system for combinatorial metabolic engineering since it combines the advantages of both quantification and modularity with the ease of programmable activation and inhibition. Various characteristics of this dual system such as Cas protein engineering, types of effectors, and RNA-riboswitches could be further improved in the near future. Nevertheless, the development of more robust multi-functional CRISPR tools to tackle complex phenotypes and enable whole-genome scale engineering remains an ongoing challenge within the context of microbial factory applications.

## Data Availability

The datasets presented in this study can be found in online repositories. The names of the repository/repositories and accession number(s) can be found in the article/Supplementary Material.
