# Supplementary material for: Quantitative and modularized CRISPR/dCas9-dCpf1 dual function system in Saccharomyces cerevisiae
Source: Front Bioeng Biotechnol. 2023 Oct 18;11:1218832. doi: 10.3389/fbioe.2023.1218832 (PMC10666755; doi:10.3389/fbioe.2023.1218832)
Supplement: Supplementary file 1 [file Table1.DOCX]

Supplementary Material

**Modularized and quantitative CRISPR/dCas9-dCpf1 dual function system in Saccharomyces cerevisiae**

**Qing Feng,1 XiaoYu Ning,1 Lei Qin,2,* Jun Li,1,* Chun Li**

1 Key Laboratory of Medical Molecule Science and Pharmaceutics Engineering, Ministry of Industry and Information Technology, School of Chemistry and Chemical Engineering, Beijing Institute of Technology, Beijing 102488, China

2 Key Lab for Industrial Biocatalysis, Ministry of Education, Department of Chemical Engineering, Tsinghua University, Beijing 100084, China

*** Correspondence:**
**Jun Li**
Email: junlibiotech@bit.edu.cn

Table S1. Plasmids and primers used for construction.

| **Strain** | **Genetype** | **sources** |
| --- | --- | --- |
| BY4741 | MATa; HIS3Δ 1; LEU2Δ 0; MET15Δ 0; URA3Δ 0 | Lab preservation |
| PUC19-dCas9 | Amp, Contains TEF1p-dCas9-CYC1t | Lab preservation |
| HCKan-T | Kan, Contains CYC1t-KanMX4-HOR | Lab preservation |
| pJZC552-scRNA(ERG7) | Amp, Contains SNR52p-gRNA -SUP4t | Lab preservation |
| pESC-Ura | Integrative vector with URA3 marker | Lab preservation |
| pESC-His | Integrative vector with HIS marker | Lab preservation |
| BY4741-01 | BY4741, ΔHO::PGK1p-dCas9-TPS1t-FBA1p-mcherry-CYC1t-KanMX4 | This study |
| BY4741-02 | BY4741, ΔHO::PGK1p-dCas9-TPS1t-FBA1p-eGFP-CYC1t-KanMX4 | This study |
| BY4741-03 | BY4741, ΔHO::PGK1p-dCas9-TPS1t-TEF1p-mcherry-CYC1t-KanMX4 | This study |
| BY4741-04 | BY4741, ΔHO::PGK1p-dCas9-TPS1t-TEF1p-eGFP-CYC1t-KanMX4 | This study |
| pESC-Ura-MCP-VP64 | Amp, Contains ADH1p-MCP-linker-VP64-ADH1t | This study |
| pESC-Ura-MCP-KRAB | Amp, Contains ADH1p-*MCP-linker-KRAB*-ADH1t | This study |
| Ura-MV-01-10 | Amp, Contains ADH1p-*MCP-linker-VP64*-ADH1t&SNR52p-*scRNA-MS2-F1-F10-*SUP4t | This study |
| Ura-MV-11-16 | Amp, Contains ADH1p-*MCP-linker-VP64*-ADH1t&SNR52p- *scRNA-MS2-T_1_1-T_1_6-*SUP4t | This study |
| Ura-MK-01-10 | Amp, Contains ADH1p-*MCP-linker-KRAB*-ADH1t&SNR52p-*scRNA-MS2-F1-F10-*SUP4t | This study |
| Ura-MK-11-16 | Amp, Contains ADH1p-*MCP-linker-KRAB*-ADH1t&SNR52p-*scRNA-MS2-T_1_1-T_1_6-*SUP4t | This study |
| Ura-01-10 | Amp, Contains SNR52p-scRNA-MS2-F1-F10-SUP4t | This study |
| Ura-11-16 | Amp, Contains SNR52p-scRNA-MS2-T11-T16-SUP4t | This study |
| BY4741-01.1-01.10 | BY4741-01:: Ura-MV-01-10 | This study |
| BY4741-01.11-01.20 | BY4741-01:: Ura-MK-01-10 | This study |
| BY4741-01.21-01.31 | BY4741-01:: Ura-01-10 | This study |
| BY4741-03.1-01.6 | BY4741-01:: Ura-MV-11-16 | This study |
| BY4741-03.7-01.12 | BY4741-01:: Ura-MK-11-16 | This study |
| BY4741-03.12-01.18 | BY4741-01:: Ura-11-16 | This study |
| BY4741-02.1-02.14 | BY4741-01:: Ura-MV/Ura-MK/Ura | This study |
| BY4741-04.1-02.12 | BY4741-01:: Ura-MV/Ura-MK/Ura | This study |
| pESC-ura-PCP-VP64 | Amp, Contains ADH1p-PCP-linker-VP64-ADH1t | This study |
| pESC-ura-PCP-KRAB | Amp, Contains ADH1p-PCP-linker-KRAB-ADH1t | This study |
| Ura-PV-01-10 | Amp, Contains ADH1p-PCP-linker-VP64-ADH1t&SNR52p- scRNA-PP7-F1-F10-SUP4t | This study |
| Ura-PV-11-16 | Amp, Contains ADH1p-PCP-linker-VP64-ADH1t&SNR52p-scRNA-PP7-T11-T16-SUP4t | This study |
| Ura-PK-01-10 | Amp, Contains ADH1p-PCP-linker-KRAB-ADH1t&SNR52p- scRNA-PP7-F1-F10-SUP4t | This study |
| Ura-PK-11-16 | Amp, Contains ADH1p-PCP-linker-KRAB-ADH1t&SNR52p-scRNA-PP7-T11-T16-SUP4t | This study |
| BY4741-01.31-01.40 | BY4741-01:: Ura-PV-01-10 | This study |
| BY4741-01.41-01.50 | BY4741-01:: Ura-PK-01-10 | This study |
| BY4741-03.19-01.24 | BY4741-01:: Ura-PV-11-16 | This study |
| BY4741-03.25-01.30 | BY4741-01:: Ura-PK-11-16 | This study |
| BY4741-02.15-02.26 | BY4741-01:: Ura-MV/Ura-MK/Ura | This study |
| BY4741-04.13-02.20 | BY4741-01:: Ura-MV/Ura-MK | This study |
| pESC-Ura-MCP-KRAB-MeCP2 | Amp, Contains ADH1p-MCP-linker-KRAB-MeCP2-ADH1t | This study |
| pESC-Ura-PCP-KRAB-MeCP2 | Amp, Contains ADH1p-PCP-linker-KRAB-MeCP2-ADH1t | This study |
| pESC-Ura-MCP-VPR | Amp, Contains ADH1p-MCP-linker-VPR-ADH1t | This study |
| pESC-Ura-PCP-VPR | Amp, Contains ADH1p-PCP-linker-VPR-ADH1t | This study |
| Ura-MCP-KRAB-MeCP2-01-10 | Amp, Contains ADH1p-MCP-linker-KRAB-MeCP2-ADH1t&SNR52p-scRNA-MS2-F1-F10-SUP4t | This study |
| Ura-MCP-KRAB-MeCP2-11-16 | Amp, Contains ADH1p-MCP-linker-KRAB-MeCP2-ADH1t&SNR52p-scRNA-MS2-T11-T16-SUP4t | This study |
| Ura-PCP-KRAB-MeCP2-01-10 | Amp, Contains ADH1p-PCP-linker-KRAB-MeCP2-ADH1t&SNR52p-scRNA-PP7-F1-F10-SUP4t | This study |
| Ura-PCP-KRAB-MeCP2-11-16 | Amp, Contains ADH1p-PCP-linker-KRAB-MeCP2-ADH1t&SNR52p-scRNA-PP7-T11-T16-SUP4t | This study |
| Ura-MCP-VPR-01-10 | Amp, Contains ADH1p-MCP-linker-VPR-ADH1t&SNR52p-scRNA-MS2-F1-F10-SUP4t | This study |
| Ura-MCP-VPR-11-16 | Amp, Contains ADH1p-MCP-linker-VPR-ADH1t&SNR52p-scRNA-MS2-T11-T16-SUP4t | This study |
| Ura-MCP-VPR-01-10 | Amp, Contains ADH1p-PCP-linker-VPR-ADH1t&SNR52p-scRNA-PP7-F1-F10-SUP4t | This study |
| Ura-MCP-VPR-11-16 | Amp, Contains ADH1p-PCP-linker-VPR-ADH1t&SNR52p-scRNA-PP7-T11-T16-SUP4t | This study |
| BY4741-01.51-01.60 | BY4741-01:: Ura-MCP-VPR-01-10 | This study |
| BY4741-01.61-01.70 | BY4741-01:: Ura-MCP-KRAB-MeCP2-01-10 | This study |
| BY4741-01.71-01.80 | BY4741-01:: Ura-PCP-VPR-01-10 | This study |
| BY4741-01.81-01.90 | BY4741-01:: Ura-PCP-KRAB-MeCP2-01-10 | This study |
| BY4741-03.31-01.36 | BY4741-01:: Ura-MCP-VPR-11-16 | This study |
| BY4741-03.37-01.42 | BY4741-01:: Ura-MCP-KRAB-MeCP2-11-16 | This study |
| BY4741-03.43-01.48 | BY4741-01:: Ura-PCP-VPR-11-16 | This study |
| BY4741-03.49-01.54 | BY4741-01:: Ura-PCP-KRAB-MeCP2-01-10 | This study |
| BY4741-02.27-02.44 | BY4741-01:: Ura-MVPR/Ura-MKM/ Ura-PVPR/Ura-PKM | This study |
| BY4741-04.21-04.36 | BY4741-01:: Ura-MVPR/Ura-MKM/ Ura-PVPR/Ura-PKM | This study |
| pY004-FnCpf1 | Amp, Contains FnCpf1 | Sichuan University |
| BY4741-05 | BY4741, ΔHO::PGK1p-dCpf1-TPS1t-FBA1p-eGFP-CYC1t-KanMX4 | This study |
| Ura-crRNA1-crRNA3 | Amp, Contains crRNA1-3 | This study |
| BY4741-05.1-05.3 | BY4741-01:: Ura-crRNA1-3 | This study |
| BY4741-06 | BY4741, ΔNTR1::PGK1p-dCpf1-TPS1t-HygR | This study |
| His-crRNA4-crRNA7 | Amp, Contains crRNA4-7 | This study |
| BY4741-06.1-06.4 | BY4741-06:: Hra-crRNA4-crRNA7 | This study |
| Ura-ecrRNA1-ecrRNA3 | Amp, Contains ecrRNA1-3 | This study |
| BY4741-05.4-05.6 | BY4741-01:: Ura-ecrRNA1-3 | This study |
| Ura-sgRNA1（eGFP） | Amp, Contains gRNA1（eGFP） | This study |
| Ura-gRNA2（eGFP） | Amp, Contains gRNA2（eGFP） | This study |
| BY4741-07 | BY4741, ΔHO::PGK1p-dCas9-TPS1t-FBA1p-mcherry-CYC1t-KanMX4&ΔNTR1::PGK1p-dCpf1-TPS1t-FBA1p-eGFP-CYC1t-HygR | This study |
| Ura-gRNA1 | Amp, Contains gRNA1 | This study |
| Ura-gRNA2 | Amp, Contains gRNA2 | This study |
| Ura-crRNA2-gRNA1 | Amp, Contains crRNA2 and gRNA1 | This study |
| Ura-crRNA2-gRNA2 | Amp, Contains crRNA2 and gRNA2 | This study |
| BY4741-07.1-07.5 | BY4741-07:: Ura-crRNA2/ Ura-gRNA1/ Ura-gRNA2/ Ura-crRNA2-gRNA1/ Ura-crRNA2-gRNA2 | This study |
| BY4741-08 | BY4741, ΔYPRC3:: TEF1p-crtE-TEF1t-FBA1p-crtI-FBA1t-TEF1p-crtYB-TEF2t-KanMX4 | This study |
| BY4741-09 | BY4741-08, ΔHO:: PGK1p-dCas9-TPS1t- Bleomycin | This study |
| delta_HAP2_HygR | Amp, Contains HygR | This study |
| delta_HAP3_BleR | Amp, Contains Bleomycin | This study |
| BY4741-10 | BY4741-8, ΔNTR1:: PGK1p-dCpf1-TPS1t-HygR | This study |
| BY4741-09.1-09.24 | BY4741-09:: Ura-MV/Ura-MK/ Ura-PV/Ura-PK/Ura-MVPR/Ura-MKM/ Ura-PVPR/Ura-PKM | This study |
| BY4741-10.1-10.4 | BY4741-10::His-crRNA4-7 | This study |
| BY471-11 | BY4741-10, ΔNTR1:: PGK1p-dCpf1-TPS1t-HygR | This study |
| BY4741-11.1-11.4 | BY4741-11:: Ura-MV-gRNA6-scRNA14/Ura-MVPR-scRNA6-gRNA14/Ura-PV-gRNA3-gRNA12/Ura-PVPR-gRNA3-gRNA2& His-crRNA7 | This study |

**Table S2.** Complementary gRNA and crRNA plasmids constructed in this study.

| RNA Plasmid | Cas9 | Target | Position | Protospacer |
| --- | --- | --- | --- | --- |
| Ura-01-1 | SpCas9 | FBA1p | -605 to -624 | CAACGTATGTGAAGCCAAGT |
| Ura-01-2 | SpCas9 | FBA1p | -458 to -477 | TTTAACGTGGGCGAAGAAGA |
| Ura-01-3 | SpCas9 | FBA1p | -482 to -501 | GTCATTACGTAAATAATGAT |
| Ura-01-4 | SpCas9 | FBA1p | -426 to -445 | TAGCAGCCGTCGGGAAAACG |
| Ura-01-5 | SpCas9 | FBA1p | -368 to -387 | ATGGAAAGAGAGGATGCTCA |
| Ura-01-6 | SpCas9 | FBA1p | -303 to-322 | GCGTTGCTCCAAAAAAGTAT |
| Ura-01-7 | SpCas9 | FBA1p | -194 to-213 | TTTAACGTGGGCGAAGAAGA |
| Ura-01-8 | SpCas9 | FBA1p | -164 to-183 | TTATCCCTCATGTTGTCTAA |
| Ura-01-9 | SpCas9 | FBA1p | -76 to-96 | AAGAACAGAAGAATAACGCA |
| Ura-01-10 | SpCas9 | FBA1p | -33 to -52 | GAATATGTATTACTTGGTTA |
| Ura-01-11 | SpCas9 | TEF1p | -485 to -504 | ATAGGAAGCAACAGGCGCGT |
| Ura-01-12 | SpCas9 | TEF1p | -435 to -454 | GTGTGGGGGATCACTTGTGG |
| Ura-01-13 | SpCas9 | TEF1p | -373 to -392 | GCGCGGAGTCCGAGAAAATC |
| Ura-01-14 | SpCas9 | TEF1p | -304 to -323 | ACGGCTGCTAGAATGGAAAA |
| Ura-01-15 | SpCas9 | TEF1p | -260 to -279 | ACACCCTAGAGGAAGAAAGA |
| Ura-01-16 | SpCas9 | TEF1p | -138 to -157 | AATTACCCGTACTAAAGGTT |
| Plasmid Cpf1 | Cpf1 | Target | Position | Protospacer |
| His-crRNA1 | FnCpf1 | ERG9p | -328 to -358 | ATCAAGGACGCCTCAATCTCG |
| His-crRNA2 | FnCpf1 | ERG9p | -248 to -268 | TATCGGGTAGAAGTTGTTGTT |
| His-crRNA3 | FnCpf1 | ERG9p | -185 to -205 | TGACGTGAAACGTAGCCTTC |
| His-crRNA4 | FnCpf1 | ERG9p | -103 to-123 | GGACAGGATTGAAAGGTAA |

**Table S3.** Oligonucleotides used in this study.

| Oligos | Sequences (5’-3’) |
| --- | --- |
| SNR52p-gKpnI-522-F | GTGACCGTGGTGGATTCGGCTCTTTGAAAAGATAATGTATGATTATG |
| SNR52p-gRNA-R | NNNNNNNNNNNNNNNNNNNNGATCATTTATCTTTCACTGC |
| gRNA -F | NNNNNNNNNNNNNNNNNNNNGTTTTAGAGCTAGAAATAGC |
| SUP4t-gKpnI-522-R | ACCCTCTTCTTGTTTTAGGGAGACATAAAAAACAAAAAAAGGGA |
| dCas9- F | ATATAAAACAATGGACAAGAAGTACTCCATTGG |
| dCas9- R | ATCGGGTTCATCACACCTTCCTCTTCTTCTTGG |
| FndCpf1-F | CAATCTAATCTAAGATGAGCATCTACCAGGAGTT |
| FndCpf1-R | TTTGCATCGGGTTCATTAGGCATAGTCGGGGACAT |
| MS2-F | GTTTTAGAGCTAGGCCAAC |
| MS2-R | GCACCACCGACTCGG |
| PP7-F | GTTTTAGAGCTAGGGCCGAAAG |
| PP7-R | AGACATAAAAAACAAAAAAAGCACCACC |
| MCP-F | ATGGCTTCTAATTTTACTCAATTTGTTTTGG |
| MCP-R | AAAGAAAAGAAAAGTTGCTGCAGCT |
| PCP-F | TCTAAAACTATTGTTTTGTCTGTTGGTGA |
| PCP-R | TTGTTAATTTGGTTCCATTGGGTAGA |
| VP64-F | CAAGAGGTAGAGCAGACGCT |
| VP64-R | TTTGGATGACTTCGATTTAGATATGTTAATTAAT |
| VPR-F | CAAGAGGTAGAGCAGACGCT |
| VPR-R | CTGGTTTGTCTATTTTTGATACTTCTTTGTTTTAA |
| KRAB-F | GTGAAGAACCATGGTTGGTTTAA |
| KRAB-R | AGAACTTTGGTTACTTTTAAAGATGTTTTTGT |
| KRAB -MeCP2-F | GTGAAGAACCATGGTTGGTTTAA |
| KRAB -MeCP2-R | CTCCTGTTACTGAAAGAGTTTCTTAA |
| MCP-KRAB-F | GCTTCTAATTTTACTCAATTTGTTTTGGTT |
| MCP-KRAB-R | AACTCCTGTTACTGAAAGAGTTTCTTAA |
| MCP-VP64-F | GCTTCTAATTTTACTCAATTTGTTTTGGTT |
| MCP-VP64-R | GGATGACTTCGATTTAGATATGTTAATTAATTAA |
| MCP-VPR-F | GCTTCTAATTTTACTCAATTTGTTTTGGTT |
| MCP-VPR-R | ACTGGTTTGTCTATTTTTGATACTTCTTTGTTTTAA |
| MCP-KM-F | GCTTCTAATTTTACTCAATTTGTTTTGGTT |
| MCP-KM-R | CTCCTGTTACTGAAAGAGTTTCTTAA |
| PCP-KRAB-F | TCTAAAACTATTGTTTTGTCTGTTGGTGA |
| PCP-KRAB-R | AACTCCTGTTACTGAAAGAGTTTCTTAA |
| PCP-VP64-F | TCTAAAACTATTGTTTTGTCTGTTGGTGA |
| PCP-VP64-R | GGATGACTTCGATTTAGATATGTTAATTAATTAA |
| PCP-VPR-F | TCTAAAACTATTGTTTTGTCTGTTGGTGA |
| PCP-VPR-R | ACTGGTTTGTCTATTTTTGATACTTCTTTGTTTTAA |
| PCP-KM-F | TCTAAAACTATTGTTTTGTCTGTTGGTGA |
| PCP-KM-R | CTCCTGTTACTGAAAGAGTTTCTTAA |
| FBA1p-F | ATAACAATACTGACAGTACTAAATAATTG |
| FBA1p-R | CCATAACCAAGTAATACATATTCAAA |
| TEF1p-F | ACAATGCATACTTTGTACGTTC |
| TEF1p-R | GCAATCTAATCTAAGTTTTAATTACAAA |
| mcherry-F | ATGGCTTCCTCCGAAGAC |
| mcherry-r | TCACTCCACCGGTGCTTAA |

**Table S4.** Oligonucleotides used to construct gRNAs and crRNAs

| Oligos | Sequences (5’-3’) |
| --- | --- |
| Ura-01-1-F | CAACGTATGTGAAGCCAAGTGTTTTAGAGCTAGAAATAGC |
| Ura-01-1-R | ACTTGGCTTCACATACGTTGGATCATTTATCTTTCACTGC |
| Ura-01-2-F | TTTAACGTGGGCGAAGAAGAGTTTTAGAGCTAGAAATAGC |
| Ura-01-2-R | TCTTCTTCGCCCACGTTAAAGATCATTTATCTTTCACTGC |
| Ura-01-3-F | GTCATTACGTAAATAATGATGTTTTAGAGCTAGAAATAGC |
| Ura-01-3-R | ATCATTATTTACGTAATGACGATCATTTATCTTTCACTGC |
| Ura-01-4-F | TAGCAGCCGTCGGGAAAACGGTTTTAGAGCTAGAAATAGC |
| Ura-01-4-R | CGTTTTCCCGACGGCTGCTAGATCATTTATCTTTCACTGC |
| Ura-01-5-F | ATGGAAAGAGAGGATGCTCAGTTTTAGAGCTAGAAATAGC |
| Ura-01-5-R | TGAGCATCCTCTCTTTCCATGATCATTTATCTTTCACTGC |
| Ura-01-6-F | GCGTTGCTCCAAAAAAGTATGTTTTAGAGCTAGAAATAGC |
| Ura-01-6-R | ATACTTTTTTGGAGCAACGCGATCATTTATCTTTCACTGC |
| Ura-01-7-F | AAGAACAGAAGAATAACGCAGTTTTAGAGCTAGAAATAGC |
| Ura-01-7-R | TCTTCTTCGCCCACGTTAAAGATCATTTATCTTTCACTGC |
| Ura-01-8-F | TTATCCCTCATGTTGTCTAAGTTTTAGAGCTAGAAATAGC |
| Ura-01-8-R | TTAGACAACATGAGGGATAAGATCATTTATCTTTCACTGC |
| Ura-01-9-F | AAGAACAGAAGAATAACGCAGTTTTAGAGCTAGAAATAGC |
| Ura-01-9-R | TGCGTTATTCTTCTGTTCTTGATCATTTATCTTTCACTGC |
| Ura-01-10-F | GAATATGTATTACTTGGTTAGTTTTAGAGCTAGAAATAGC |
| Ura-01-10-R | TAACCAAGTAATACATATTCGATCATTTATCTTTCACTGC |
| Ura-01-11-F | ATAGGAAGCAACAGGCGCGTGTTTTAGAGCTAGAAATAGC |
| Ura-01-11-R | ACGCGCCTGTTGCTTCCTATGATCATTTATCTTTCACTGC |
| Ura-01-12-F | GTGTGGGGGATCACTTGTGGGTTTTAGAGCTAGAAATAGC |
| Ura-01-12-R | CCACAAGTGATCCCCCACACGATCATTTATCTTTCACTGC |
| Ura-01-13-F | GCGCGGAGTCCGAGAAAATCGTTTTAGAGCTAGAAATAGC |
| Ura-01-13-R | GATTTTCTCGGACTCCGCGCGATCATTTATCTTTCACTGC |
| Ura-01-14-F | ACACCCTAGAGGAAGAAAGAGTTTTAGAGCTAGAAATAGC |
| Ura-01-14-R | TCTTTCTTCCTCTAGGGTGTGATCATTTATCTTTCACTGC |
| Ura-01-15-F | AATTACCCGTACTAAAGGTTGTTTTAGAGCTAGAAATAGC |
| Ura-01-15-R | AACCTTTAGTACGGGTAATTGATCATTTATCTTTCACTGC |
| Ura-01-16-F | TTAACTTAAATATCAATGGGGTTTTAGAGCTAGAAATAGC |
| Ura-01-16-R | CCCATTGATATTTAAGTTAAGATCATTTATCTTTCACTGC |
| His-crRNA1-F | GTGACCGTGGTGGATTCGGCTCTTTGAAAAGATAATGTATGATTATG |
| His-crRNA1-R | CATAAAAAACAAAAAAAGCTCTAACTCCGCAGGAACTAATCTACCATAGTAGAAATTG |
| His-crRNA2-F | GTGACCGTGGTGGATTCGGCTCTTTGAAAAGATAATGTATGATTATG |
| His-crRNA2-R | CATAAAAAACAAAAAAATTGTTGTTGAAGATGGGCTATATCTACCATAGTAGAAATTG |
| His-crRNA3-F | GTGACCGTGGTGGATTCGGCTCTTTGAAAAGATAATGTATGATTATG |
| His-crRNA3-R | CATAAAAAACAAAAAAACTTCCGATGCAAAGTGCAGTGATCTACCATAGTAGAAATTG |
| His-crRNA4-F | GTGACCGTGGTGGATTCGGCTCTTTGAAAAGATAATGTATGATTATG |
| His-crRNA4-R | CATAAAAAACAAAAAAAAATGGAAAGTTAGGACAGGGGATCTACCATAGTAGAAATTG |

**Table S5.** Sequences of the DNA fragments used in this work

**Templates for the design of gRNA oligos:**

FW:5’-**GCAGTGAAAGATAAATGATCNNNNNNNNNNNNNNNNNNNNGTTTTAGAGCTA** RW:5’-**GAAATAGCCTAGCTCTAAAACNNNNNNNNNNNNNNNNNNNNGATCATTTATC**

**Sp-gRNA scaffold** DNA fragment:

GTTTTAGAGCTAGAAATAGCAAGTTAAAATAAGGCTAGTCCGTTATCAACTTGAAAAAGTGGCACCGAGTCGGTGC

**Sp-gRNA-2XMS2 scaffold** DNA fragment:

GTTTTAGAGCTAGGCCAACATGAGGATCACCCATGTCTGCAGGGCCTAGCAAGTTAAAATAAGGCTAGTCCGTTATCAACTTGGCCAACATGAGGATCACCCATGTCTGCAGGGCCAAGTGGCACCGAGTCGGTGGTGC

**Sp-gRNA-2XPP7 scaffold** DNA fragment:

GTTTTAGAGCTAGGGCCGAAAGGAGCAGACGATATGGCGTCGCTCCCTGCGGCCTAGCAAGTTAAAATAAGGCTAGTCCGTTATCAACTTGGGCCGAAAGGAGCAGACGATATGGCGTCGCTCCCTGCGGCCAAGTGGCACCGAGTCGGTGGTGCTTTTTTTGTTTTTTATGTCT

**Sp-dCas9 DNA fragment**

atggacaagaagtactccattgggctcgctatcggcacaaacagcgtcggttgggccgtcattacggacgagtacaaggtgccgagcaaaaaattcaaagttctgggcaataccgatcgccacagcataaagaagaacctcattggcgccctcctgttcgactccggggagacggccgaagccacgcggctcaaaagaacagcacggcgcagatatacccgcagaaagaatcggatctgctacctgcaggagatctttagtaatgagatggctaaggtggatgactctttcttccataggctggaggagtcctttttggtggaggaggataaaaagcacgagcgccacccaatctttggcaatatcgtggacgaggtggcgtaccatgaaaagtacccaaccatatatcatctgaggaagaagcttgtagacagtactgataaggctgacttgcggttgatctatctcgcgctggcgcatatgatcaaatttcggggacacttcctcatcgagggggacctgaacccagacaacagcgatgtcgacaaactctttatccaactggttcagacttacaatcagcttttcgaagagaacccgatcaacgcatccggagttgacgccaaagcaatcctgagcgctaggctgtccaaatcccggcggctcgaaaacctcatcgcacagctccctggggagaagaagaacggcctgtttggtaatcttatcgccctgtcactcgggctgacccccaactttaaatctaacttcgacctggccgaagatgccaagcttcaactgagcaaagacacctacgatgatgatctcgacaatctgctggcccagatcggcgaccagtacgcagacctttttttggcggcaaagaacctgtcagacgccattctgctgagtgatattctgcgagtgaacacggagatcaccaaagctccgctgagcgctagtatgatcaagcgctatgatgagcaccaccaagacttgactttgctgaaggcccttgtcagacagcaactgcctgagaagtacaaggaaattttcttcgatcagtctaaaaatggctacgccggatacattgacggcggagcaagccaggaggaattttacaaatttattaagcccatcttggaaaaaatggacggcaccgaggagctgctggtaaagcttaacagagaagatctgttgcgcaaacagcgcactttcgacaatggaagcatcccccaccagattcacctgggcgaactgcacgctatcctcaggcggcaagaggatttctacccctttttgaaagataacagggaaaagattgagaaaatcctcacatttcggataccctactatgtaggccccctcgcccggggaaattccagattcgcgtggatgactcgcaaatcagaagagaccatcactccctggaacttcgaggaagtcgtggataagggggcctctgcccagtccttcatcgaaaggatgactaactttgataaaaatctgcctaacgaaaaggtgcttcctaaacactctctgctgtacgagtacttcacagtttataacgagctcaccaaggtcaaatacgtcacagaagggatgagaaagccagcattcctgtctggagagcagaagaaagctatcgtggacctcctcttcaagacgaaccggaaagttaccgtgaaacagctcaaagaagactatttcaaaaagattgaatgtttcgactctgttgaaatcagcggagtggaggatcgcttcaacgcatccctgggaacgtatcacgatctcctgaaaatcattaaagacaaggacttcctggacaatgaggagaacgaggacattcttgaggacattgtcctcacccttacgttgtttgaagatagggagatgattgaagaacgcttgaaaacttacgctcatctcttcgacgacaaagtcatgaaacagctcaagaggcgccgatatacaggatgggggcggctgtcaagaaaactgatcaatgggatccgagacaagcagagtggaaagacaatcctggattttcttaagtccgatggatttgccaaccggaacttcatgcagttgatccatgatgactctctcacctttaaggaggacatccagaaagcacaagtttctggccagggggacagtcttcacgagcacatcgctaatcttgcaggtagcccagctatcaaaaagggaatactgcagaccgttaaggtcgtggatgaactcgtcaaagtaatgggaaggcataagcccgagaatatcgttatcgagatggcccgagagaaccaaactacccagaagggacagaagaacagtagggaaaggatgaagaggattgaagagggtataaaagaactggggtcccaaatccttaaggaacacccagttgaaaacacccagcttcagaatgagaagctctacctgtactacctgcagaacggcagggacatgtacgtggatcaggaactggacatcaatcggctctccgactacgacgtggatGCTatcgtgccccagtcttttctcaaagatgattctattgataataaagtgttgacaagatccgataaaaatagagggaagagtgataacgtcccctcagaagaagttgtcaagaaaatgaaaaattattggcggcagctgctgaacgccaaactgatcacacaacggaagttcgataatctgactaaggctgaacgaggtggcctgtctgagttggataaagccggcttcatcaaaaggcagcttgttgagacacgccagatcaccaagcacgtggcccaaattctcgattcacgcatgaacaccaagtacgatgaaaatgacaaactgattcgagaggtgaaagttattactctgaagtctaagctggtctcagatttcagaaaggactttcagttttataaggtgagagagatcaacaattaccaccatgcgcatgatgcctacctgaatgcagtggtaggcactgcacttatcaaaaaatatcccaagcttgaatctgaatttgtttacggagactataaagtgtacgatgttaggaaaatgatcgcaaagtctgagcaggaaataggcaaggccaccgctaagtacttcttttacagcaatattatgaattttttcaagaccgagattacactggccaatggagagattcggaagcgaccacttatcgaaacaaacggagaaacaggagaaatcgtgtgggacaagggtagggatttcgcgacagtccggaaggtcctgtccatgccgcaggtgaacatcgttaaaaagaccgaagtacagaccggaggcttctccaaggaaagtatcctcccgaaaaggaacagcgacaagctgatcgcacgcaaaaaagattgggaccccaagaaatacggcggattcgattctcctacagtcgcttacagtgtactggttgtggccaaagtggagaaagggaagtctaaaaaactcaaaagcgtcaaggaactgctgggcatcacaatcatggagcgatcaagcttcgaaaaaaaccccatcgactttctcgaggcgaaaggatataaagaggtcaaaaaagacctcatcattaagcttcccaagtactctctctttgagcttgaaaacggccggaaacgaatgctcgctagtgcgggcgagctgcagaaaggtaacgagctggcactgccctctaaatacgttaatttcttgtatctggccagccactatgaaaagctcaaagggtctcccgaagataatgagcagaagcagctgttcgtggaacaacacaaacactaccttgatgagatcatcgagcaaataagcgaattctccaaaagagtgatcctcgccgacgctaacctcgataaggtgctttctgcttacaataagcacagggataagcccatcagggagcaggcagaaaacattatccacttgtttactctgaccaacttgggcgcgcctgcagccttcaagtacttcgacaccaccatagacagaaagcggtacacctctacaaaggaggtcctggacgccacactgattcatcagtcaattacggggctctatgaaacaagaatcgacctctctcagctcggtggagac

**Fn-dCpf1 DNA fragment**

atgagcatctaccaggagttcgtcaacaagtattcactgagtaagacactgcggttcgagctgatcccacagggcaagacactggagaacatcaaggcccgaggcctgattctggacgatgagaagcgggcaaaagactataagaaagccaagcagatcattgataaataccaccagttctttatcgaggaaattctgagctccgtgtgcatcagtgaggatctgctgcagaattactcagacgtgtacttcaagctgaagaagagcgacgatgacaacctgcagaaggacttcaagtccgccaaggacaccatcaagaaacagattagcgagtacatcaaggactccgaaaagtttaaaaatctgttcaaccagaatctgatcgatgctaagaaaggccaggagtccgacctgatcctgtggctgaaacagtctaaggacaatgggattgaactgttcaaggctaactccgatatcactgatattgacgaggcactggaaatcatcaagagcttcaagggatggaccacatactttaaaggcttccacgagaaccgcaagaacgtgtactccagcaacgacattcctacctccatcatctaccgaatcgtcgatgacaatctgccaaagttcctggagaacaaggccaaatatgaatctctgaaggacaaagctcccgaggcaattaattacgaacagatcaagaaagatctggctgaggaactgacattcgatatcgactataagactagcgaggtgaaccagagggtcttttccctggacgaggtgtttgaaatcgccaatttcaacaattacctgaaccagtccggcattactaaattcaataccatcattggcgggaagtttgtgaacggggagaataccaagcgcaagggaattaacgaatacatcaatctgtatagccagcagatcaacgacaaaactctgaagaaatacaagatgtctgtgctgttcaaacagatcctgagtgataccgagtccaagtcttttgtcattgataaactggaagatgactcagacgtggtcactaccatgcagagcttttatgagcagatcgccgctttcaagacagtggaggaaaaatctattaaggaaactctgagtctgctgttcgatgacctgaaagcccagaagctggacctgagtaagatctacttcaaaaacgataagagtctgacagacctgtcacagcaggtgtttgatgactattccgtgattgggaccgccgtcctggagtacattacacagcagatcgctccaaagaacctggataatccctctaagaaagagcaggaactgatcgctaagaaaaccgagaaggcaaaatatctgagtctggaaacaattaagctggcactggaggagttcaacaagcacagggatattgacaaacagtgccgctttgaggaaatcctggccaacttcgcagccatccccatgatttttgatgagatcgcccagaacaaagacaatctggctcagatcagtattaagtaccagaaccagggcaagaaagacctgctgcaggcttcagcagaagatgacgtgaaagccatcaaggatctgctggaccagaccaacaatctgctgcacaagctgaaaatcttccatattagtcagtcagaggataaggctaatatcctggataaagacgaacacttctacctggtgttcgaggaatgttacttcgagctggcaaacattgtccccctgtataacaagattaggaactacatcacacagaagccttactctgacgagaagtttaaactgaacttcgaaaatagtaccctggccaacgggtgggataagaacaaggagcctgacaacacagctatcctgttcatcaaggatgacaagtactatctgggagtgatgaataagaaaaacaataagatcttcgatgacaaagccattaaggagaacaaaggggaaggatacaagaaaatcgtgtataagctgctgcccggcgcaaataagatgctgcctaaggtgttcttcagcgccaagagtatcaaattctacaacccatccgaggacatcctgcggattagaaatcactcaacacatactaagaacgggagcccccagaagggatatgagaaatttgagttcaacatcgaggattgcaggaagtttattgacttctacaagcagagcatctccaaacaccctgaatggaaggattttggcttccggttttccgacacacagagatataactctatcgacgagttctaccgcgaggtggaaaatcaggggtataagctgacttttgagaacatttctgaaagttacatcgacagcgtggtcaatcagggaaagctgtacctgttccagatctataacaaagatttttcagcatacagcaagggcagaccaaacctgcatacactgtactggaaggccctgttcgatgagaggaatctgcaggacgtggtctataaactgaacggagaggccgaactgttttaccggaagcagtctattcctaagaaaatcactcacccagctaaggaggccatcgctaacaagaacaaggacaatcctaagaaagagagcgtgttcgaatacgatctgattaaggacaagcggttcaccgaagataagttctttttccattgtccaatcaccattaacttcaagtcaagcggcgctaacaagttcaacgacgagatcaatctgctgctgaaggaaaaagcaaacgatgtgcacatcctgagcattGCCcgaggagagcggcatctggcctactataccctggtggatggcaaagggaatatcattaagcaggatacattcaacatcattggcaatgaccggatgaaaaccaactaccacgataaactggctgcaatcgagaaggatagagactcagctaggaaggactggaagaaaatcaacaacattaaggagatgaaggaaggctatctgagccaggtggtccatgagattgcaaagctggtcatcgaatacaatgccattgtggtgttcgaggatctgaacttcggctttaagagggggcgctttaaggtggaaaaacaggtctatcagaagctggagaaaatgctgatcgaaaagctgaattacctggtgtttaaagataacgagttcgacaagaccggaggcgtcctgagagcctaccagctgacagctccctttgaaactttcaagaaaatgggaaaacagacaggcatcatctactatgtgccagccggattcacttccaagatctgccccgtgaccggctttgtcaaccagctgtaccctaaatatgagtcagtgagcaagtcccaggaatttttcagcaagttcgataagatctgttataatctggacaaggggtacttcgagttttccttcgattacaagaacttcggcgacaaggccgctaaggggaaatggaccattgcctccttcggatctcgcctgatcaactttcgaaattccgataaaaaccacaattgggacactagggaggtgtacccaaccaaggagctggaaaagctgctgaaagactactctatcgagtatggacatggcgaatgcatcaaggcagccatctgtggcgagagtgataagaaatttttcgccaagctgacctcagtgctgaatacaatcctgcagatgcggaactcaaagaccgggacagaactggactatctgattagccccgtggctgatgtcaacggaaacttcttcgacagcagacaggcacccaaaaatatgcctcaggatgcagacgccaacggggcctaccacatcgggctgaagggactgatgctgctgggccggatcaagaacaatcaggaggggaagaagctgaacctggtcattaagaacgaggaatacttcgagtttgtccagaatagaaataacaaaaggccggcggccacgaaaaaggccggccaggcaaaaaagaaaaagggatcctacccatacgatgttccagattacgcttatccctacgacgtgcctgattatgcatacccatatgatgtccccgactatgcctaa

**MCP DNA fragment**

ATGGCTTCTAATTTTACTCAATTTGTTTTGGTTGATAATGGTGGTACTGGTGATGTTACTGTTGCTCCATCTAATTTTGCTAATGGTGTTGCTGAATGGATTTCTTCTAATTCTAGATCTCAAGCTTATAAAGTTACTTGTTCTGTCAGACAATCTTCTGCTCAAAAAAGAAAATATACTATTAAAGTTGAAGTTCCAAAAGTTGCTACTCAAACTGTTGGTGGTGTTGAATTGCCTGTTGCTGCTTGGAGATCTTATTTGAATATGGAATTGACTATTCCAATTTTTGCTACTAATTCTGATTGTGAATTGATTGTTAAAGCTATGCAAGGTTTGTTGAAAGATGGTAATCCAATTCCATCTGCTATTGCTGCTAATTCTGGTATTTATTCTGCTGGTGGAGGTGGTTCTGGTGGAGGTGGTTCTGGTGGAGGTGGTTCTGGACCAAAAAAGAAAAGAAAAGTTGCTGCAGCT

**PCP DNA fragment**

ATGTCTAAAACTATTGTTTTGTCTGTTGGTGAAGCTACTAGAACTTTGACTGAAATTCAATCTACTGCTGATAGACAAATTTTTGAAGAAAAAGTTGGTCCATTGGTTGGTAGATTGAGATTGACTGCTTCTTTGAGACAAAATGGTGCTAAAACTGCTTATAGAGTTAATTTGAAATTGGATCAAGCTGATGTTGTTGATTCTGGTTTGCCAAAAGTTAGATATACTCAAGTTTGGTCTCATGATGTTACTATTGTTGCTAATTCTACTGAAGCTTCTAGAAAATCTTTGTATGATTTGACTAAATCTTTGGTTGCTACTTCTCAAGTTGAAGATTTGGTTGTTAATTTGGTTCCATTGGGTAGATCTGCTGGTGGAGGTGGTTCTGGTGGAGGTGGTTCTGGTGGTGGTGGTTCTGGTCCAAAAAAGAAAAGAAAAGTTGCTGCAGCT

**VP64 DNA fragment**

CCAAGAGGTAGAGCAGACGCTTTGGATGATTTCGATTTGGATATGTTAGGTTCAGACGCATTGGATGACTTTGATTTAGATATGTTGGGTTCTGATGCTTTAGATGATTTCGATTTGGATATGTTGGGTTCTGATGCTTTGGATGACTTCGATTTAGATATGTTAATTAAT

**P65 DNA fragment**

GCTTCTAGATCTTCTGGTTCACCAAAAAAGAAAAGAAAAGTTGGTTCTCAATATTTGCCTGATACTGATGATAGGCATAGGATCGAAGAAAAGAGAAAAAGGACTTATGAAACTTTTAAATCAATTATGAAAAAATCTCCATTTTCTGGTCCAACTGATCCAAGACCACCTCCAAGGAGAATTGCTGTTCCATCTAGATCTTCTGCTTCTGTTCCAAAACCTGCTCCACAACCATATCCATTTACTTCTTCTTTGTCTACTATTAATTATGATGAATTTCCAACTATGGTTTTTCCATCTGGTCAAATTTCTCAAGCTTCAGCTTTGGCTCCTGCTCCACCACAAGTTTTGCCACAAGCTCCTGCTCCTGCTCCTGCTCCTGCTATGGTTTCTGCATTAGCTCAAGCTCCTGCTCCTGTTCCTGTTTTAGCTCCTGGTCCACCACAAGCTGTTGCTCCACCTGCTCCAAAACCAACTCAAGCTGGTGAAGGTACTTTGTCTGAAGCTTTGTTGCAATTGCAATTTGATGATGAAGATTTGGGTGCTTTGTTGGGTAACTCTACTGATCCTGCTGTTTTTACTGATTTGGCTTCTGTTGATAATTCTGAATTTCAACAATTGTTGAATCAAGGTATTCCTGTTGCTCCACATACTACTGAACCAATGTTGATGGAATATCCTGAAGCTATTACTAGATTGGTTACTGGTGCTCAAAGACCACCTGATCCTGCTCCTGCTCCATTGGGTGCTCCTGGTTTGCCAAATGGTTTGTTGTCTGGTGATGAAGATTTTTCTTCTATTGCTGATATGGATTTTTCTGCTTTGTTGGGTTCTGGATCTGGTTCT

**Rta DNA fragment**

AGAGATTCTAGAGAAGGTATGTTTTTGCCAAAACCTGAAGCTGGTTCTGCTATTTCTGATGTTTTTGAAGGTAGAGAAGTTTGTCAACCAAAAAGAATTAGACCATTTCATCCACCTGGTTCTCCATGGGCTAATAGACCATTGCCTGCTTCTTTGGCTCCAACTCCAACTGGTCCTGTTCATGAACCTGTTGGTTCTTTGACTCCTGCTCCTGTTCCTCAACCATTGGATCCTGCTCCTGCAGTTACACCTGAAGCTTCTCATTTGTTGGAAGATCCTGATGAAGAAACTTCTCAAGCTGTTAAAGCTTTGAGAGAAATGGCTGATACTGTTATTCCACAAAAAGAAGAAGCTGCTATTTGTGGTCAAATGGATTTGTCTCATCCACCTCCAAGAGGACATTTGGATGAATTGACTACAACTTTGGAATCTATGACTGAAGATTTGAATTTGGATTCTCCATTGACTCCTGAATTGAATGAAATTTTGGATACTTTTTTGAATGATGAATGTTTGTTGCATGCTATGCATATTTCTACTGGTTTGTCTATTTTTGATACTTCTTTGTTTTAA

**VPR DNA fragment**

CCAAGAGGTAGAGCAGACGCTTTGGATGATTTCGATTTGGATATGTTAGGTTCAGACGCATTGGATGACTTTGATTTAGATATGTTGGGTTCTGATGCTTTAGATGATTTCGATTTGGATATGTTGGGTTCTGATGCTTTGGATGACTTCGATTTAGATATGTTAATTAATGCTTCTAGATCTTCTGGTTCACCAAAAAAGAAAAGAAAAGTTGGTTCTCAATATTTGCCTGATACTGATGATAGGCATAGGATCGAAGAAAAGAGAAAAAGGACTTATGAAACTTTTAAATCAATTATGAAAAAATCTCCATTTTCTGGTCCAACTGATCCAAGACCACCTCCAAGGAGAATTGCTGTTCCATCTAGATCTTCTGCTTCTGTTCCAAAACCTGCTCCACAACCATATCCATTTACTTCTTCTTTGTCTACTATTAATTATGATGAATTTCCAACTATGGTTTTTCCATCTGGTCAAATTTCTCAAGCTTCAGCTTTGGCTCCTGCTCCACCACAAGTTTTGCCACAAGCTCCTGCTCCTGCTCCTGCTCCTGCTATGGTTTCTGCATTAGCTCAAGCTCCTGCTCCTGTTCCTGTTTTAGCTCCTGGTCCACCACAAGCTGTTGCTCCACCTGCTCCAAAACCAACTCAAGCTGGTGAAGGTACTTTGTCTGAAGCTTTGTTGCAATTGCAATTTGATGATGAAGATTTGGGTGCTTTGTTGGGTAACTCTACTGATCCTGCTGTTTTTACTGATTTGGCTTCTGTTGATAATTCTGAATTTCAACAATTGTTGAATCAAGGTATTCCTGTTGCTCCACATACTACTGAACCAATGTTGATGGAATATCCTGAAGCTATTACTAGATTGGTTACTGGTGCTCAAAGACCACCTGATCCTGCTCCTGCTCCATTGGGTGCTCCTGGTTTGCCAAATGGTTTGTTGTCTGGTGATGAAGATTTTTCTTCTATTGCTGATATGGATTTTTCTGCTTTGTTGGGTTCTGGATCTGGTTCTAGAGATTCTAGAGAAGGTATGTTTTTGCCAAAACCTGAAGCTGGTTCTGCTATTTCTGATGTTTTTGAAGGTAGAGAAGTTTGTCAACCAAAAAGAATTAGACCATTTCATCCACCTGGTTCTCCATGGGCTAATAGACCATTGCCTGCTTCTTTGGCTCCAACTCCAACTGGTCCTGTTCATGAACCTGTTGGTTCTTTGACTCCTGCTCCTGTTCCTCAACCATTGGATCCTGCTCCTGCAGTTACACCTGAAGCTTCTCATTTGTTGGAAGATCCTGATGAAGAAACTTCTCAAGCTGTTAAAGCTTTGAGAGAAATGGCTGATACTGTTATTCCACAAAAAGAAGAAGCTGCTATTTGTGGTCAAATGGATTTGTCTCATCCACCTCCAAGAGGACATTTGGATGAATTGACTACAACTTTGGAATCTATGACTGAAGATTTGAATTTGGATTCTCCATTGACTCCTGAATTGAATGAAATTTTGGATACTTTTTTGAATGATGAATGTTTGTTGCATGCTATGCATATTTCTACTGGTTTGTCTATTTTTGATACTTCTTTGTTTTAA

**KRAB DNA fragment**

AGAACTTTGGTTACTTTTAAAGATGTTTTTGTTGATTTTACTAGAGAAGAATGGAAATTGTTGGATACTGCACAACAAATCGTTTATAGAAATGTTATGTTGGAAAATTATAAAAATTTGGTTTCATTAGGTTATCAATTGACTAAACCTGATGTTATTTTGAGATTGGAAAAAGGTGAAGAACCATGGTTGGTT

**MeCP2 DNA fragment**

TCTGGTGGAGGTTCTGGTGGTTCTGGTTCTTCTCCAAAAAAGAAAAGAAAAGTTGAAGCTTCTGTTCAAGTTAAAAGAGTTTTGGAAAAATCACCTGGTAAATTGTTGGTTAAAATGCCATTTCAAGCTTCTCCTGGTGGTAAAGGTGAAGGTGGAGGTGCTACTACTTCTGCTCAAGTTATGGTTATTAAAAGACCTGGTAGAAAAAGAAAGGCTGAAGCTGATCCACAAGCTATTCCAAAAAAAAGAGGTAGAAAACCTGGTTCTGTTGTTGCTGCAGCTGCAGCTGAAGCTAAAAAGAAAGCTGTTAAAGAATCTTCTATTAGATCTGTTCAAGAAACAGTCTTACCAATTAAAAAAAGAAAAACAAGAGAAACTGTTTCTATCGAAGTCAAAGAAGTTGTTAAACCATTGTTGGTTTCTACTTTGGGTGAAAAATCTGGTAAAGGTTTGAAAACTTGTAAATCTCCTGGAAGAAAATCAAAAGAATCTTCTCCAAAAGGTAGATCTTCATCTGCTTCTTCTCCACCAAAAAAAGAACATCACCATCATCACCATCATGCTGAGTCTCCAAAAGCTCCAATGCCATTGTTGCCACCTCCACCTCCACCTGAACCACAATCATCTGAAGATCCAATTTCTCCACCTGAACCTCAAGATTTGTCTTCATCTATTTGTAAAGAAGAAAAAATGCCAAGAGCTGGTTCTTTGGAATCTGATGGTTGTCCAAAAGAACCTGCTAAAACTCAACCAATGGTTGCAGCTGCAGCTACTACAACTACTACAACTACAACTACTGTTGCTGAAAAATATAAACATAGAGGTGAAGGTGAAAGAAAAGATATTGTTTCTTCATCTATGCCAAGACCAAATAGAGAAGAACCTGTTGATTCTAGAACTCCTGTTACTGAAAGAGTTTCTTAA

**KRAB-MeCP2 (KM) DNA fragment**

AGAACTTTGGTTACTTTTAAAGATGTTTTTGTTGATTTTACTAGAGAAGAATGGAAATTGTTGGATACTGCACAACAAATCGTTTATAGAAATGTTATGTTGGAAAATTATAAAAATTTGGTTTCATTAGGTTATCAATTGACTAAACCTGATGTTATTTTGAGATTGGAAAAAGGTGAAGAACCATGGTTGGTTTCTGGTGGAGGTTCTGGTGGTTCTGGTTCTTCTCCAAAAAAGAAAAGAAAAGTTGAAGCTTCTGTTCAAGTTAAAAGAGTTTTGGAAAAATCACCTGGTAAATTGTTGGTTAAAATGCCATTTCAAGCTTCTCCTGGTGGTAAAGGTGAAGGTGGAGGTGCTACTACTTCTGCTCAAGTTATGGTTATTAAAAGACCTGGTAGAAAAAGAAAGGCTGAAGCTGATCCACAAGCTATTCCAAAAAAAAGAGGTAGAAAACCTGGTTCTGTTGTTGCTGCAGCTGCAGCTGAAGCTAAAAAGAAAGCTGTTAAAGAATCTTCTATTAGATCTGTTCAAGAAACAGTCTTACCAATTAAAAAAAGAAAAACAAGAGAAACTGTTTCTATCGAAGTCAAAGAAGTTGTTAAACCATTGTTGGTTTCTACTTTGGGTGAAAAATCTGGTAAAGGTTTGAAAACTTGTAAATCTCCTGGAAGAAAATCAAAAGAATCTTCTCCAAAAGGTAGATCTTCATCTGCTTCTTCTCCACCAAAAAAAGAACATCACCATCATCACCATCATGCTGAGTCTCCAAAAGCTCCAATGCCATTGTTGCCACCTCCACCTCCACCTGAACCACAATCATCTGAAGATCCAATTTCTCCACCTGAACCTCAAGATTTGTCTTCATCTATTTGTAAAGAAGAAAAAATGCCAAGAGCTGGTTCTTTGGAATCTGATGGTTGTCCAAAAGAACCTGCTAAAACTCAACCAATGGTTGCAGCTGCAGCTACTACAACTACTACAACTACAACTACTGTTGCTGAAAAATATAAACATAGAGGTGAAGGTGAAAGAAAAGATATTGTTTCTTCATCTATGCCAAGACCAAATAGAGAAGAACCTGTTGATTCTAGAACTCCTGTTACTGAAAGAGTTTCTTAA

**RecVect-Amp ::Receiver vector for gRNAs assembly platform**

tcgcgcgtttcggtgatgacggtgaaaacctctgacacatgcagctcccggagacggtcacagcttgtctgtaagcggatgccgggagcagacaagcccgtcagggcgcgtcagcgggtgttggcgggtgtcggggctggcttaactatgcggcatcagagcagattgtactgagagtgcaccataccacagcttttcaattcaattcatcattttttttttattcttttttttgatttcggtttctttgaaatttttttgattcggtaatctccgaacagaaggaagaacgaaggaaggagcacagacttagattggtatatatacgcatatgtagtgttgaagaaacatgaaattgcccagtattcttaacccaactgcacagaacaaaaacctgcaggaaacgaagataaatcatgtcgaaagctacatataaggaacgtgctgctactcatcctagtcctgttgctgccaagctatttaatatcatgcacgaaaagcaaacaaacttgtgtgcttcattggatgttcgtaccaccaaggaattactggagttagttgaagcattaggtcccaaaatttgtttactaaaaacacatgtggatatcttgactgatttttccatggagggcacagttaagccgctaaaggcattatccgccaagtacaattttttactcttcgaagacagaaaatttgctgacattggtaatacagtcaaattgcagtactctgcgggtgtatacagaatagcagaatgggcagacattacgaatgcacacggtgtggtgggcccaggtattgttagcggtttgaagcaggcggcagaagaagtaacaaaggaacctagaggccttttgatgttagcagaattgtcatgcaagggctccctatctactggagaatatactaagggtactgttgacattgcgaagagcgacaaagattttgttatcggctttattgctcaaagagacatgggtggaagagatgaaggttacgattggttgattatgacacccggtgtgggtttagatgacaagggagacgcattgggtcaacagtatagaaccgtggatgatgtggtctctacaggatctgacattattattgttggaagaggactatttgcaaagggaagggatgctaaggtagagggtgaacgttacagaaaagcaggctgggaagcatatttgagaagatgcggccagcaaaactaaaaaactgtattataagtaaatgcatgtatactaaactcacaaattagagcttcaatttaattatatcagttattaccctatgcggtgtgaaataccgcacagatgcgtaaggagaaaataccgcatcaggaaattgtaaacgttaatattttgttaaaattcgcgttaaatttttgttaaatcagctcattttttaaccaataggccgaaatcggcaaaatcccttataaatcaaaagaatagaccgagatagggttgagtgttgttccagtttggaacaagagtccactattaaagaacgtggactccaacgtcaaagggcgaaaaaccgtctatcagggcgatggcccactacgtgaaccatcaccctaatcaagttttttggggtcgaggtgccgtaaagcactaaatcggaaccctaaagggagcccccgatttagagcttgacggggaaagccggcgaacgtggcgagaaaggaagggaagaaagcgaaaggagcgggcgctagggcgctggcaagtgtagcggtcacgctgcgcgtaaccaccacacccgccgcgcttaatgcgccgctacagggcgcgtccattcgccattcaggctgcgcaactgttgggaagggcgatcggtgcgggcctcttcgctattacgccagctgaattggagcgacctcatgctatacctgagaaagcaacctgacctacaggaaagagttactcaagaataagaattttcgttttaaaacctaagagtcactttaaaatttgtatacacttattttttttataacttatttaataataaaaatcataaatcataagaaattcgcttatttagaagtgtcaacaacgtatctaccaacgatttgacccttttccatcttttcgtaaatttctggcaaggtagacaagccgacaaccttgattggagacttgaccaaacctctggcgaagaattgttaattaagagctcagatcttatcgtcgtcatccttgtaatccatcgatactagtgcggccgccctttagtgagggttgaattcgaattttcaaaaattcttactttttttttggatggacgcaaagaagtttaataatcatattacatggcattaccaccatatacatatccatatacatatccatatctaatcttacttatatgttgtggaaatgtaaagagccccattatcttagcctaaaaaaaccttctctttggaactttcagtaatacgcttaactgctcattgctatattgaagtacggattagaagccgccgagcgggtgacagccctccgaaggaagactctcctccgtgcgtcctcgtcttcaccggtcgcgttcctgaaacgcagatgtgcctcgcgccgcactgctccgaacaataaagattctacaatactagcttttatggttatgaagaggaaaaattggcagtaacctggccccacaaaccttcaaatgaacgaatcaaattaacaaccataggatgataatgcgattagttttttagccttatttctggggtaattaatcagcgaagcgatgatttttgatctattaacagatatataaatgcaaaaactgcataaccactttaactaatactttcaacattttcggtttgtattacttcttattcaaatgtaataaaagtatcaacaaaaaattgttaatatacctctatactttaacgtcaaggagaaaaaaccccggatccgtaatacgactcactatagggcccgggcgtcgacatggaacagaagttgatttccgaagaagacctcgagtaagcttggtaccgcggctagctaagatccgctctaaccgaaaaggaaggagttagacaacctgaagtctaggtccctatttatttttttatagttatgttagtattaagaacgttatttatatttcaaatttttcttttttttctgtacagacgcgtgtacgcatgtaacattatactgaaaaccttgcttgagaaggttttgggacgctcgaagatccagctgcattaatgaatcggccaacgcgcggggagaggcggtttgcgtattgggcgctcttccgcttcctcgctcactgactcgctgcgctcggtcgttcggctgcggcgagcggtatcagctcactcaaaggcggtaatacggttatccacagaatcaggggataacgcaggaaagaacatgtgagcaaaaggccagcaaaaggccaggaaccgtaaaaaggccgcgttgctggcgtttttccataggctccgcccccctgacgagcatcacaaaaatcgacgctcaagtcagaggtggcgaaacccgacaggactataaagataccaggcgtttccccctggaagctccctcgtgcgctctcctgttccgaccctgccgcttaccggatacctgtccgcctttctcccttcgggaagcgtggcgctttctcatagctcacgctgtaggtatctcagttcggtgtaggtcgttcgctccaagctgggctgtgtgcacgaaccccccgttcagcccgaccgctgcgccttatccggtaactatcgtcttgagtccaacccggtaagacacgacttatcgccactggcagcagccactggtaacaggattagcagagcgaggtatgtaggcggtgctacagagttcttgaagtggtggcctaactacggctacactagaaggacagtatttggtatctgcgctctgctgaagccagttaccttcggaaaaagagttggtagctcttgatccggcaaacaaaccaccgctggtagcggtggtttttttgtttgcaagcagcagattacgcgcagaaaaaaaggatctcaagaagatcctttgatcttttctacggggtctgacgctcagtggaacgaaaactcacgttaagggattttggtcatgagattatcaaaaaggatcttcacctagatccttttaaattaaaaatgaagttttaaatcaatctaaagtatatatgagtaaacttggtctgacagttaccaatgcttaatcagtgaggcacctatctcagcgatctgtctatttcgttcatccatagttgcctgactccccgtcgtgtagataactacgatacgggagggcttaccatctggccccagtgctgcaatgataccgcgagacccacgctcaccggctccagatttatcagcaataaaccagccagccggaagggccgagcgcagaagtggtcctgcaactttatccgcctccatccagtctattaattgttgccgggaagctagagtaagtagttcgccagttaatagtttgcgcaacgttgttgccattgctacaggcatcgtggtgtcacgctcgtcgtttggtatggcttcattcagctccggttcccaacgatcaaggcgagttacatgatcccccatgttgtgcaaaaaagcggttagctccttcggtcctccgatcgttgtcagaagtaagttggccgcagtgttatcactcatggttatggcagcactgcataattctcttactgtcatgccatccgtaagatgcttttctgtgactggtgagtactcaaccaagtcattctgagaatagtgtatgcggcgaccgagttgctcttgcccggcgtcaatacgggataataccgcgccacatagcagaactttaaaagtgctcatcattggaaaacgttcttcggggcgaaaactctcaaggatcttaccgctgttgagatccagttcgatgtaacccactcgtgcacccaactgatcttcagcatcttttactttcaccagcgtttctgggtgagcaaaaacaggaaggcaaaatgccgcaaaaaagggaataagggcgacacggaaatgttgaatactcatactcttcctttttcaatattattgaagcatttatcagggttattgtctcatgagcggatacatatttgaatgtatttagaaaaataaacaaataggggttccgcgcacatttccccgaaaagtgccacctgaacgaagcatctgtgcttcattttgtagaacaaaaatgcaacgcgagagcgctaatttttcaaacaaagaatctgagctgcatttttacagaacagaaatgcaacgcgaaagcgctattttaccaacgaagaatctgtgcttcatttttgtaaaacaaaaatgcaacgcgagagcgctaatttttcaaacaaagaatctgagctgcatttttacagaacagaaatgcaacgcgagagcgctattttaccaacaaagaatctatacttcttttttgttctacaaaaatgcatcccgagagcgctatttttctaacaaagcatcttagattactttttttctcctttgtgcgctctataatgcagtctcttgataactttttgcactgtaggtccgttaaggttagaagaaggctactttggtgtctattttctcttccataaaaaaagcctgactccacttcccgcgtttactgattactagcgaagctgcgggtgcattttttcaagataaaggcatccccgattatattctataccgatgtggattgcgcatactttgtgaacagaaagtgatagcgttgatgattcttcattggtcagaaaattatgaacggtttcttctattttgtctctatatactacgtataggaaatgtttacattttcgtattgttttcgattcactctatgaatagttcttactacaatttttttgtctaaagagtaatactagagataaacataaaaaatgtagaggtcgagtttagatgcaagttcaaggagcgaaaggtggatgggtaggttatatagggatatagcacagagatatatagcaaagagatacttttgagcaatgtttgtggaagcggtattcgcaatattttagtagctcgttacagtccggtgcgtttttggttttttgaaagtgcgtcttcagagcgcttttggttttcaaaagcgctctgaagttcctatactttctagagaataggaacttcggaataggaacttcaaagcgtttccgaaaacgagcgcttccgaaaatgcaacgcgagctgcgcacatacagctcactgttcacgtcgcacctatatctgcgtgttgcctgtatatatatatacatgagaagaacggcatagtgcgtgtttatgcttaaatgcgtacttatatgcgtctatttatgtaggatgaaaggtagtctagtacctcctgtgatattatcccattccatgcggggtatcgtatgcttccttcagcactaccctttagctgttctatatgctgccactcctcaattggattagtctcatccttcaatgctatcatttcctttgatattggatcatactaagaaaccattattatcatgacattaacctataaaaataggcgtatcacgaggccctttcgtc

**RecVect-Amp ::Receiver vector for crRNAs assembly platform**

tcgcgcgtttcggtgatgacggtgaaaacctctgacacatgcagctcccggagacggtcacagcttgtctgtaagcggatgccgggagcagacaagcccgtcagggcgcgtcagcgggtgttggcgggtgtcggggctggcttaactatgcggcatcagagcagattgtactgagagtgcaccataaattcccgttttaagagcttggtgagcgctaggagtcactgccaggtatcgtttgaacacggcattagtcagggaagtcataacacagtcctttcccgcaattttctttttctattactcttggcctcctctagtacactctatatttttttatgcctcggtaatgattttcatttttttttttcccctagcggatgactctttttttttcttagcgattggcattatcacataatgaattatacattatataaagtaatgtgatttcttcgaagaatatactaaaaaatgagcaggcaagataaacgaaggcaaagatgacagagcagaaagccctagtaaagcgtattacaaatgaaaccaagattcagattgcgatctctttaaagggtggtcccctagcgatagagcactcgatcttcccagaaaaagaggcagaagcagtagcagaacaggccacacaatcgcaagtgattaacgtccacacaggtatagggtttctggaccatatgatacatgctctggccaagcattccggctggtcgctaatcgttgagtgcattggtgacttacacatagacgaccatcacaccactgaagactgcgggattgctctcggtcaagcttttaaagaggccctactggcgcgtggagtaaaaaggtttggatcaggatttgcgcctttggatgaggcactttccagagcggtggtagatctttcgaacaggccgtacgcagttgtcgaacttggtttgcaaagggagaaagtaggagatctctcttgcgagatgatcccgcattttcttgaaagctttgcagaggctagcagaattaccctccacgttgattgtctgcgaggcaagaatgatcatcaccgtagtgagagtgcgttcaaggctcttgcggttgccataagagaagccacctcgcccaatggtaccaacgatgttccctccaccaaaggtgttcttatgtagtgacaccgattatttaaagctgcagcatacgatatatatacatgtgtatatatgtatacctatgaatgtcagtaagtatgtatacgaacagtatgatactgaagatgacaaggtaatgcatcattctatacgtgtcattctgaacgaggcgcgctttccttttttctttttgctttttctttttttttctcttgaactcgacggatctatgcggtgtgaaataccgcacagatgcgtaaggagaaaataccgcatcaggaaattgtaaacgttaatattttgttaaaattcgcgttaaatttttgttaaatcagctcattttttaaccaataggccgaaatcggcaaaatcccttataaatcaaaagaatagaccgagatagggttgagtgttgttccagtttggaacaagagtccactattaaagaacgtggactccaacgtcaaagggcgaaaaaccgtctatcagggcgatggcccactacgtgaaccatcaccctaatcaagttttttggggtcgaggtgccgtaaagcactaaatcggaaccctaaagggagcccccgatttagagcttgacggggaaagccggcgaacgtggcgagaaaggaagggaagaaagcgaaaggagcgggcgctagggcgctggcaagtgtagcggtcacgctgcgcgtaaccaccacacccgccgcgcttaatgcgccgctacagggcgcgtcgcgccattcgccattcaggctgcgcaactgttgggaagggcgatcggtgcgggcctcttcgctattacgccagctgaattggagcgacctcatgctatacctgagaaagcaacctgacctacaggaaagagttactcaagaataagaattttcgttttaaaacctaagagtcactttaaaatttgtatacacttattttttttataacttatttaataataaaaatcataaatcataagaaattcgcttatttagaagtgtcaacaacgtatctaccaacgatttgacccttttccatcttttcgtaaatttctggcaaggtagacaagccgacaaccttgattggagacttgaccaaacctctggcgaagaattgttaattaagagctcagatcttatcgtcgtcatccttgtaatccatcgatactagtgcggccgccctttagtgagggttgaattcgaattttcaaaaattcttactttttttttggatggacgcaaagaagtttaataatcatattacatggcattaccaccatatacatatccatatacatatccatatctaatcttacttatatgttgtggaaatgtaaagagccccattatcttagcctaaaaaaaccttctctttggaactttcagtaatacgcttaactgctcattgctatattgaagtacggattagaagccgccgagcgggtgacagccctccgaaggaagactctcctccgtgcgtcctcgtcttcaccggtcgcgttcctgaaacgcagatgtgcctcgcgccgcactgctccgaacaataaagattctacaatactagcttttatggttatgaagaggaaaaattggcagtaacctggccccacaaaccttcaaatgaacgaatcaaattaacaaccataggatgataatgcgattagttttttagccttatttctggggtaattaatcagcgaagcgatgatttttgatctattaacagatatataaatgcaaaaactgcataaccactttaactaatactttcaacattttcggtttgtattacttcttattcaaatgtaataaaagtatcaacaaaaaattgttaatatacctctatactttaacgtcaaggagaaaaaaccccggatccgtaatacgactcactatagggcccgggcgtcgacatggaacagaagttgatttccgaagaagacctcgagtaagcttggtaccgcggctagctaagatccgctctaaccgaaaaggaaggagttagacaacctgaagtctaggtccctatttatttttttatagttatgttagtattaagaacgttatttatatttcaaatttttcttttttttctgtacagacgcgtgtacgcatgtaacattatactgaaaaccttgcttgagaaggttttgggacgctcgaagatccagctgcattaatgaatcggccaacgcgcggggagaggcggtttgcgtattgggcgctcttccgcttcctcgctcactgactcgctgcgctcggtcgttcggctgcggcgagcggtatcagctcactcaaaggcggtaatacggttatccacagaatcaggggataacgcaggaaagaacatgtgagcaaaaggccagcaaaaggccaggaaccgtaaaaaggccgcgttgctggcgtttttccataggctccgcccccctgacgagcatcacaaaaatcgacgctcaagtcagaggtggcgaaacccgacaggactataaagataccaggcgtttccccctggaagctccctcgtgcgctctcctgttccgaccctgccgcttaccggatacctgtccgcctttctcccttcgggaagcgtggcgctttctcatagctcacgctgtaggtatctcagttcggtgtaggtcgttcgctccaagctgggctgtgtgcacgaaccccccgttcagcccgaccgctgcgccttatccggtaactatcgtcttgagtccaacccggtaagacacgacttatcgccactggcagcagccactggtaacaggattagcagagcgaggtatgtaggcggtgctacagagttcttgaagtggtggcctaactacggctacactagaaggacagtatttggtatctgcgctctgctgaagccagttaccttcggaaaaagagttggtagctcttgatccggcaaacaaaccaccgctggtagcggtggtttttttgtttgcaagcagcagattacgcgcagaaaaaaaggatctcaagaagatcctttgatcttttctacggggtctgacgctcagtggaacgaaaactcacgttaagggattttggtcatgagattatcaaaaaggatcttcacctagatccttttaaattaaaaatgaagttttaaatcaatctaaagtatatatgagtaaacttggtctgacagttaccaatgcttaatcagtgaggcacctatctcagcgatctgtctatttcgttcatccatagttgcctgactccccgtcgtgtagataactacgatacgggagggcttaccatctggccccagtgctgcaatgataccgcgagacccacgctcaccggctccagatttatcagcaataaaccagccagccggaagggccgagcgcagaagtggtcctgcaactttatccgcctccatccagtctattaattgttgccgggaagctagagtaagtagttcgccagttaatagtttgcgcaacgttgttgccattgctacaggcatcgtggtgtcacgctcgtcgtttggtatggcttcattcagctccggttcccaacgatcaaggcgagttacatgatcccccatgttgtgcaaaaaagcggttagctccttcggtcctccgatcgttgtcagaagtaagttggccgcagtgttatcactcatggttatggcagcactgcataattctcttactgtcatgccatccgtaagatgcttttctgtgactggtgagtactcaaccaagtcattctgagaatagtgtatgcggcgaccgagttgctcttgcccggcgtcaatacgggataataccgcgccacatagcagaactttaaaagtgctcatcattggaaaacgttcttcggggcgaaaactctcaaggatcttaccgctgttgagatccagttcgatgtaacccactcgtgcacccaactgatcttcagcatcttttactttcaccagcgtttctgggtgagcaaaaacaggaaggcaaaatgccgcaaaaaagggaataagggcgacacggaaatgttgaatactcatactcttcctttttcaatattattgaagcatttatcagggttattgtctcatgagcggatacatatttgaatgtatttagaaaaataaacaaataggggttccgcgcacatttccccgaaaagtgccacctgaacgaagcatctgtgcttcattttgtagaacaaaaatgcaacgcgagagcgctaatttttcaaacaaagaatctgagctgcatttttacagaacagaaatgcaacgcgaaagcgctattttaccaacgaagaatctgtgcttcatttttgtaaaacaaaaatgcaacgcgagagcgctaatttttcaaacaaagaatctgagctgcatttttacagaacagaaatgcaacgcgagagcgctattttaccaacaaagaatctatacttcttttttgttctacaaaaatgcatcccgagagcgctatttttctaacaaagcatcttagattactttttttctcctttgtgcgctctataatgcagtctcttgataactttttgcactgtaggtccgttaaggttagaagaaggctactttggtgtctattttctcttccataaaaaaagcctgactccacttcccgcgtttactgattactagcgaagctgcgggtgcattttttcaagataaaggcatccccgattatattctataccgatgtggattgcgcatactttgtgaacagaaagtgatagcgttgatgattcttcattggtcagaaaattatgaacggtttcttctattttgtctctatatactacgtataggaaatgtttacattttcgtattgttttcgattcactctatgaatagttcttactacaatttttttgtctaaagagtaatactagagataaacataaaaaatgtagaggtcgagtttagatgcaagttcaaggagcgaaaggtggatgggtaggttatatagggatatagcacagagatatatagcaaagagatacttttgagcaatgtttgtggaagcggtattcgcaatattttagtagctcgttacagtccggtgcgtttttggttttttgaaagtgcgtcttcagagcgcttttggttttcaaaagcgctctgaagttcctatactttctagagaataggaacttcggaataggaacttcaaagcgtttccgaaaacgagcgcttccgaaaatgcaacgcgagctgcgcacatacagctcactgttcacgtcgcacctatatctgcgtgttgcctgtatatatatatacatgagaagaacggcatagtgcgtgtttatgcttaaatgcgtacttatatgcgtctatttatgtaggatgaaaggtagtctagtacctcctgtgatattatcccat

ccatgcggggtatcgtatgcttccttcagcactaccctttagctgttctatatgctgccactcctcaattggattagtctcatccttcaatgctatcatttcctttgatattggatcatctaagaaaccattattatcatgacattaacctataaaaataggcgtatcacgaggccctttc
